# How environment affects active particle swarms: a case study

**DOI:** 10.1098/rsos.220791

**Published:** 2022-12-14

**Authors:** Pierre Degond, Angelika Manhart, Sara Merino-Aceituno, Diane Peurichard, Lorenzo Sala

**Affiliations:** ^1^ Institut de Mathématiques de Toulouse, UMR5219, Université de Toulouse, CNRS, UPS, Toulouse Cedex 9 31062, France; ^2^ Mathematics Department, University College London, 25 Gordon Street, London, UK; ^3^ Faculty of Mathematics, University of Vienna, Oskar-Morgenstern-Platz 1, Vienna 1090, Austria; ^4^ Inria, Laboratoire Jacques-Louis Lions, Sorbonne Université, CNRS, Université de Paris, 4, Place Jussieu, Paris Cedex 05 75252, France; ^5^ INRIA Saclay Ile-de-France, 1 rue Honoré d’Estienne d’Orves, Palaiseau 91120, France

**Keywords:** self-propelled particles, collective dynamics, agent-based models, partial differential equations, multiscale modelling

## Abstract

We investigate the collective motion of self-propelled agents in an environment filled with obstacles that are tethered to fixed positions via springs. The active particles are able to modify the environment by moving the obstacles through repulsion forces. This creates feedback interactions between the particles and the obstacles from which a breadth of patterns emerges (trails, band, clusters, honey-comb structures, etc.). We will focus on a discrete model first introduced in Aceves-Sanchez P *et al.* (2020, *Bull. Math. Biol.* **82,** 125 (doi:10.1007/s11538-020-00805-z)), and derived into a continuum PDE model. As a first major novelty, we perform an in-depth investigation of pattern formation of the discrete and continuum models in two dimensions: we provide phase-diagrams and determine the key mechanisms for bifurcations to happen using linear stability analysis. As a result, we discover that the agent-agent repulsion, the agent-obstacle repulsion and the obstacle’s spring stiffness are the key forces in the appearance of patterns, while alignment forces between the particles play a secondary role. The second major novelty lies in the development of an innovative methodology to compare discrete and continuum models that we apply here to perform an in-depth analysis of the agreement between the discrete and continuum models.

## 1. Introduction

Understanding how patterns in collective motion arise from local interactions between individuals is an exciting and challenging endeavour that has drawn the attention of the scientific community [1–8]. In many scenarios, the environment plays a key role in the emergence of collective motion and of the resulting patterns [9–14]. Examples are evacuation dynamics in the presence of obstacles [6,15,16], sperm dynamics in the seminal fluid [14,17], swirl of fish under the presence of predators [18], cells moving in a space filled with fibres [11] or over a substrate [7], etc.

In particular, we are interested in feedback interactions between self-propelled agents and their environment that they are able to modify. This happens, for example, (i) in the formation of paths in grass-land by active walkers [19,20], (ii) in the modification of the extracellular matrix (fibres) by migratory cells [21] or (iii) in ant trail formation due to ant pheromone deposition [4]. In this paper, we will focus on the model introduced in [22] where collective motion happens in an environment filled with movable obstacles that are tethered to a fixed point via a spring. The authors in [22] showed that a variety of patterns are generated due to the feedback interactions between the obstacles and the self-propelled agents. Indeed, the capacity of the agents to modify their environment (i.e. to modify the position of the obstacles) is key for patterns to form.

**Figure 1.**
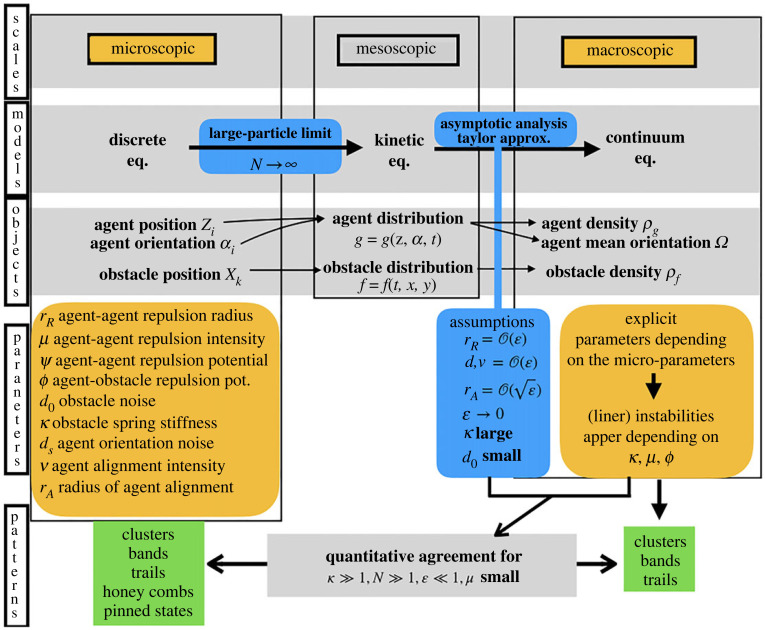
Overview of the paper. It includes a summary of the scales, the models and the objects considered in this paper and introduced in [22] (first three grey lines). The blue boxes indicate the derivation of the different models and derivation assumptions. The main contributions in the paper appear in the last row corresponding to ‘patterns’ (at the discrete and continuum level and their correspondence) and the linear stability analysis (bottom right yellow box).

Figure 1 offers an overview of the ideas and messages of this paper. We will consider mostly two scales (marked in yellow). The reason for this is that understanding the emergent properties of collective dynamics requires us to establish a link between the agent’s interactions and the continuum dynamics that emerges at scales much larger than the size of the individual agents. As a consequence, it is natural to consider two different scales to investigate collective motion: a microscopic scale where the discrete dynamics of the agents can be described, and a macroscopic scale where the average/continuum behaviour of the large ensemble can be observed.

From a modelling perspective, it is natural to consider the microscopic scale, where individual-based models can describe individual-agent behaviour and their interactions. In the left column of figure 1, we present key features of the individual-based model introduced in [22]. The model assumes that agents move trying to avoid obstacles via a repulsion force. Agents interact with each other following Vicsek-type dynamics [23–26], i.e. they move at a constant speed trying to align their orientation of motion with one of their neighbours, up to some noise, while repelling each other at short distances. The discrete system gives the time-evolution of the position of the obstacles ${\left(X_{i}\right)}_{i=1,\ldots ,N}$ tethered at fixed anchor points ${\left(Y_{i}\right)}_{i=1,\ldots ,N}$ via a spring and the position and orientation of the self-propelled agents ${\left(Z_{k},\alpha_{k}\right)}_{k=1,\ldots ,M}$, where $X_{i},Y_{i},Z_{k}\in R^{2}$ and *α*_*k*_ is a unit vector (see equation (2.1) for a full mathematical description of the system and figure 1 for a list of the most relevant parameters). We will explore the variety of patterns that arise depending on the values of the model parameters.

However, the simulation of the discrete model becomes quickly computationally challenging for systems composed of millions of individuals. Therefore, for large-particle systems, continuum models are to be preferred since they provide information on the average behaviour and are computationally less costly (right column of figure 1). Moreover, continuum models are the appropriate framework for studying large-scale patterns and carrying out mathematical analyses like linear stability analysis. The drawback is that, from a modelling perspective, they are harder to justify than individual based models. For this reason, one would like to derive the continuum dynamics from the discrete ones: this derivation validates the continuum models and provides understanding on the emergence of large-scale patterns. At the same time, during this derivation process, due to averaging and asymptotic analysis, some information on the discrete system can be lost.

This rigorous derivation is precisely one of the purposes of kinetic theory. The kinetic theory has been successfully applied to the study of models like the Vicsek model [23–26] and the Cucker–Smale model [27–29]. Tools from kinetic theory were applied in [22] to the discrete model described above, see second and third rows in figure 1.

First, the authors derive the mean-field limit equation (large-particle limit *N*, *M* → ∞ for both agents and obstacles). This equation corresponds to a Kolmogorov–Fokker–Plank equation for the time-evolution of the distribution of the agents *g* = *g*(*z*, *α*, *t*) at position $z\in R^{2}$ and orientation *α*; and the time-evolution of the distribution of the obstacles *f* = *f*(*x*, *y*, *t*) at position $x\in R^{2}$ with anchor point at $y\in R^{2}$.

Then, from the kinetic equations for these distributions, the authors in [22] obtained continuum equations for the system under some asymptotic assumptions on the parameters (right blue boxes in figure 1). In particular, a high stiffness of the obstacle springs, strong local agent-agent repulsion and fast agent alignment is assumed. In this regime, it was shown in [22] that the obstacle density *ρ*_*f*_ = *ρ*_*f*_(*x*, *t*) becomes a non-local function of the agent density *ρ*_*g*_ = *ρ*_*g*_(*x*, *t*) and that the continuum model consists of a system of two nonlinear non-local equations for *ρ*_*g*_ and the local mean orientation of the agents Ω=Ω(x,t), see equations (2.6).

The main objective of this article is to investigate the influence of the tethered obstacles in pattern formation using the discrete and continuum models first introduced in [22]. The main contributions of this paper are listed below:

— we focus our study primarily on the continuum equations (which were analysed only in dimension one in [22]). Here, we introduce two-dimensional simulations of the continuum equations and an extensive phase diagram (§3.2) that shows the appearance of patterns depending on the value of the parameters (green box in figure 1). We carry out a linear stability analysis in two dimensions around uniform states and validate this analysis by comparing its predictions with the numerical simulations of the discrete and continuum models (right yellow box in figure 1);
— we document in which parameter regime the continuum equations capture the discrete patterns (bottom grey box in figure 1). To this end, we propose a method to compare discrete and continuum simulations. This novel method provides an indicator of the distance between different patterns;
— lastly, we also expand and greatly systematize the parameter exploration of the discrete model supported by a phase diagram. As a consequence, we detect two new patterns with respect to [22]: honeycombs structures and pinned agents states (left green box in figure 1).

### 1.1. Organization of the paper

The paper is organized as follows: we first describe the models (discrete and continuum), including the derivation assumptions of the continuum model. Then we simulate both systems to construct two corresponding phase diagrams based on different values of the parameters. Next, to better understand pattern formation as a function of the model parameters, we perform a linear stability analysis of the continuum equations around uniform states and identify bifurcation parameters controlling the formation of patterns. Finally, an innovative method is proposed to compare discrete and continuum simulations, which is used to determine in which parameter regime the continuum equations are in good accordance with the discrete dynamics. We conclude the paper with a discussion of the main results.

## 2. Modelling

### 2.1. Discrete dynamics

We consider as a starting point the model introduced in [22] for self-propelled particles undergoing collective motion in an environment filled with obstacles. Obstacles are tethered to a given fixed anchor point through a Hookean spring. They are characterized by their positions $X_{i}(t)\in R^{2}$ over time *t* ≥ 0 and their anchor points $Y_{i}\in R^{2}$ for *i* = 1, 2, …, *N*, where *N* is the total number of obstacles. The self-propelled particles are characterized by their positions $Z_{k}(t)\in R^{2}$ and orientations $\alpha_{k}(t)\in S^{1}$ (unit circle) at time *t* ≥ 0, *k* = 1, 2, …, *M*, where *M* is the total number of agents. We assume that obstacles and agents interact through a given potential, as explained next.

The evolution for the obstacles ${\left(X_{i}(t),Y_{i}\right)}_{i=1,\ldots ,N}$ and the agents ${\left(Z_{k}(t),\alpha_{k}(t)\right)}_{k=1,\ldots ,M}$ over time is given by the following coupled system of stochastic differential equations:

2.1a $$dX_{i}=-\frac{\kappa }{\eta }(X_{i}-Y_{i})\;dt-\frac{1}{\eta }\frac{1}{M}\sum_{k=1}^{M}\nabla \phi (X_{i}-Z_{k})\;dt+\sqrt{2d_{o}}\;dB_{t}^{i},$$

2.1b $$\begin{array}{rl} dZ_{k} & =u_{0}\alpha_{k}\;dt-\frac{1}{\zeta }\frac{1}{N}\sum_{i=1}^{N}\nabla \phi (Z_{k}-X_{i})\;dt-\frac{1}{\zeta }\frac{1}{M}\sum_{l\neq k}^{M}\nabla \psi (Z_{k}-Z_{l})\;dt \end{array}$$

2.1c $$\mathrm{and}\;d\alpha_{k}=P_{\alpha_{k}^{⊥}}\circ \left[\nu {\bar{\alpha }}_{k}\;dt+\sqrt{2d_{s}}\;d{\tilde{B}}_{t}^{k}\right],$$

where the mean direction ${\bar{\alpha }}_{k}$ is defined via the mean flux *J*_*k*_ as follows:

2.2 $${\bar{\alpha }}_{k}=\frac{J_{k}}{\left|J_{k}\right|},\;\text{where }J_{k}=\sum_{\begin{array}{c} \;j=1\\ |Z_{k}-Z_{j}|\leq r_{A} \end{array}}^{M}\alpha_{j}.$$

Equation (2.1*a*) gives the time-evolution for the obstacles’ positions *X*_*i*_. The first term on the right-hand side corresponds to the force generated by the Hookean spring anchored at position *Y*_*i*_ with stiffness constant *κ* > 0. The tether positions *Y*_*i*_ are given and do not change over time. The terms *B*^*i*^, i=1,…,N are independent Brownian motions that introduce noise in the dynamics with intensity *d*_0_ > 0. This term accounts for fluctuations in the dynamics. Finally, the second term on the right-hand side of equation (2.1*a*) is precisely the interaction force that couples the dynamics of the self-propelled agents with those of the obstacles. We assume that *ϕ* is an even and non-negative interaction potential. Typically, we will assume *ϕ* to be a repulsive potential to model volume exclusion between obstacles and self-propelled particles.

Now, equation (2.1*b*) gives the time-evolution for the position of the self-propelled agents *Z*_*k*_. The first term on the right-hand side of (2.1*b*) expresses that agent *k* moves in the orientation *α*_*k*_ at a fixed speed *u*_0_ > 0. The second term is the force due to the interaction potential coupling the self-propelled agents and the obstacles, as we have seen before. Finally, the last term is a repulsive force between agents given by a potential *ψ* which is assumed to be non-negative and even. This force is added to the model to prevent agents clustering at a single point in space and represents volume exclusion interactions between the agents [5].

The last equation (2.1*c*) gives the time-evolution for the orientation of the agents and corresponds to the terms appearing in the Vicsek model [30], which is a widely used model in collective motion. The right-hand side of equation (2.1*c*) is the sum of two competing forces: a force that tries to align the orientation of the self-propelled agents with the mean orientation of their neighbours and a noise term that opposes this alignment. The noise is given by ${\left({\tilde{B}}^{k}\right)}_{k=1,\ldots ,M}$ which are *M* independent Brownian motions (also assumed to be independent from *B*^*i*^, i=1,…,N) and the intensity of this noise is given by the parameter *d*_*s*_ > 0. The operator $P_{\alpha_{k}^{⊥}}$ represents the orthonormal projection onto $\alpha_{k}^{⊥}$ (where $\alpha_{k}^{⊥}$ is a vector orthogonal to *α*_*k*_) and the symbol ‘°’ indicates that the stochastic differential equation has to be understood in the Stratonovich sense [31]. In particular, the projection ensures that, for all times where the dynamics are defined, *α*_*k*_(*t*) remains on the sphere, i.e. |*α*_*k*_| = 1. The alignment force is given by $P_{\alpha_{k}^{⊥}}\nu {\bar{\alpha }}_{k}$ where *ν* > 0 is a positive constant and ${\bar{\alpha }}_{k}$ is the average orientation of the neighbouring agents that are at distance *r*_*A*_ > 0 from agent *k*, as computed in equation (2.2). Indeed, this term corresponds to an alignment force since it can be rewritten as

$$P_{\alpha_{k}^{⊥}}\nu {\bar{\alpha }}_{k}=\nu \nabla_{\alpha_{k}}(\alpha_{k}\cdot {\bar{\alpha }}_{k}),$$

where $\nabla_{\alpha_{k}}$ denotes the gradient on the sphere. Therefore, this term is a gradient flow that relaxes *α*_*k*_ towards the average orientation ${\bar{\alpha }}_{k}$ at speed *ν* > 0.

Finally, note that the discrete model (2.1) consists of first-order equations: the model can be derived from second-order equations in the overdamped (or inertialess) regime. This is the reason why the parameters *η* > 0 and *ζ* > 0 appear in the system: *η* corresponds to the obstacle friction and *ζ* to the agent friction. In an inertialess regime first-order equations give a good approximation of the dynamics and this regime appears in many biological applications, in particular involving micro-agents (like sperm cells) in highly viscous environments.

As we will see in later sections, the feedback interactions between agents and between agents and obstacles give rise to a variety of patterns depending on the value of the parameters.

### 2.2. Continuum dynamics

When the number of agents and obstacles becomes large, it is useful to derive equations that determine the average behaviour of the discrete system (2.1). These ‘averaged’ equations correspond to continuum equations, which were derived in [22] for the discrete system (2.1). In this section, we summarize the results from this reference.

#### 2.2.1. Main assumptions of the derivation

The derivation of the continuum equations in [22] is done under the following set of assumptions:

**(a) Large-particle system assumption.** The number of obstacles and agents are assumed to tend to infinity, i.e. *N* → ∞, *M* → ∞.

Under this assumption, the authors derived formally equations for the evolution of obstacles and agent density (kinetic equations). Then, some of the parameters of the kinetic equation are scaled by a small factor ε≪1, and the continuum equations are obtained in the limit ε→0. We explain next the scaling assumptions considered.

**(b) Scaling assumptions on the parameters.** Three types of scaling assumptions are made

(i) the radius of alignment of the agents is supposed to be small and scaled as $r_{A}=O(\sqrt{\varepsilon })$;
(ii) the agent-agent repulsion distance is supposed to be small and scales as $r_{R}=O(\varepsilon )$, but it is ensured that the potential stays of order 1 by setting
  2.3 ∫ψ(x) dx=μ<∞;
(iii) the agents alignment rate *ν* and orientational noise intensity *d*_*s*_ in (2.1*c*) are supposed to be very large and scale as: $d_{s},\nu =O(1/\varepsilon )$ with $d_{s}/\nu =O(1)$: this corresponds to fast agent-agent alignment and diffusion [30].

**(c) Uniform anchor density and stiff regime assumptions.** It is assumed that the anchor density for the obstacles is constant (uniformly distributed) and that the obstacles’ springs are very stiff (the parameter *κ* is very large). To this end, we consider the ratio

2.4 $$\gamma =\frac{\eta }{\kappa }\ll 1$$

to be small. We suppose also a low obstacle noise regime by considering the smallness of

2.5 $$\delta =d_{o}\gamma \ll 1.$$

The set of assumptions (a) is sufficient to derive continuum equations. The large-particle-limit or mean-field limit gives rise to kinetic equations for the obstacle density *f* = *f*(*t*, *x*, *y*) and the agent density *g* = *g*(*t*, *z*, *α*). The set of assumptions (b) and (c) are sufficient to obtain closed equations for the obstacle density *ρ*_*f*_ = *ρ*_*f*_(*t*, *x*), the agent density *ρ*_*g*_ = *ρ*_*g*_(*x*, *t*) and the mean-agent orientation Ω=Ω(x,t). In particular, the scaling assumptions $r_{A}=O(\sqrt{\varepsilon })$ and $r_{R}=O(\varepsilon )$ imply that alignment and agent-agent repulsion forces become localized in space as ε→0. The set of assumptions (c) is used to Taylor expand the function *f* with respect to *γ* and *δ*.

In summary, the continuum equations approximate a system with a very large number of agents and obstacles in the regime where the parameters of the system reach a given range of values, as described above, i.e. in the regime ε→0 (by an asymptotic analysis) and *γ* ≈ 0, *δ* ≈ 0 (by a Taylor expansion approximation). These approximations will be taken into account when comparing discrete and continuum simulations, since they determine the range of validity of the continuum dynamics.

#### 2.2.2. The continuum model

The authors in [22] obtain the following equations for the dynamics of the density of agents $\rho_{g}(x,t)\in R$ and their mean orientation $\Omega (x,t)\in S^{1}$ at a point $x\in R^{2}$ at time *t* ≥ 0

2.6 $$\begin{array}{cc} & \partial_{t}\rho_{g}+\nabla \cdot (U\rho_{g})=0\\ \mathrm{and}\; & \rho_{g}\partial_{t}\Omega +\rho_{g}(V\cdot \nabla )\Omega +d_{3}P_{\Omega^{⊥}}\nabla \rho_{g}=\gamma_{s}P_{\Omega^{⊥}}\Delta (\rho_{g}\Omega ), \end{array}\}$$

where

$$U=d_{1}\Omega -\frac{1}{\zeta }\nabla {\bar{\rho }}_{f}-\frac{\mu }{\zeta }\nabla \rho_{g}$$

and

$$V=d_{2}\Omega -\frac{1}{\zeta }\nabla {\bar{\rho }}_{f}-\frac{\mu }{\zeta }\nabla \rho_{g},$$

where *ρ*_*f*_(*x*, *t*) is the obstacle density given by

2.7 $$\frac{\rho_{f}}{\rho_{A}}=1+\frac{1}{\kappa }\Delta {\bar{\rho }}_{g}+\frac{1}{\kappa^{2}}N({\bar{\rho }}_{g})-\frac{\eta }{\kappa^{2}}\partial_{t}\Delta {\bar{\rho }}_{g}+O\left({\left(\frac{\eta }{\kappa }\right)}^{3}\right),\;N({\bar{\rho }}_{g})\;:\;=\text{det}H({\bar{\rho }}_{g}),$$

where *ρ*_*A*_ is the distribution of the anchor points in space (assumed to be constant and here taken to be equal to 1 in the simulations and computations); H denotes the Hessian, ‘det’ denotes the determinant, and we have defined

2.8 $$\bar{\rho }\;:\;=\rho *\phi ,$$

the convolution between *ρ* and *ϕ*, where *ϕ* is the repulsion kernel between agents and obstacles, equation (3.1). In the numerical simulations, we will drop the higher order terms in *η*/*κ* for *ρ*_*f*_. The model parameters are the friction constants *ζ*, *η*, the obstacle-spring constant *κ* and the agent-agent repulsion intensity *μ* given by equation (2.3).

The friction coefficient *γ*_*s*_ reads

2.9 $$\gamma_{s}=\frac{r_{A}^{2}}{8}\left(\frac{d_{s}}{\nu }+c_{2}\right).$$

The constants *d*_1_, *d*_2_ and *d*_3_ are defined by

2.10 $$d_{i}=u_{0}c_{i},$$

where *u*_0_ is the agent speed, and *c*_1_, *c*_2_ and *c*_3_ are explicit constants that depend only on the fraction *d*_*s*_/*ν*

2.11a $$c_{1}=\int_{0}^{2\pi }\cos\theta m(\theta )\;d\theta ,$$

2.11b $$c_{2}=\frac{\int_{0}^{\pi }\sin^{2}\theta \cos\theta m(\theta )h(\theta )\;d\theta }{\int_{0}^{\pi }\sin^{2}\theta m(\theta )h(\theta )\;d\theta }$$

2.11c $$\mathrm{and}\;\;c_{3}=\frac{d_{s}}{\nu },$$

where

$$m(\theta )=\frac{1}{Z}\exp\left(\frac{\nu }{d_{s}}\cos\theta \right)\;\mathrm{and}\;Z\;:\;=\int_{0}^{2\pi }\exp\left(\frac{\nu \cos\theta }{d_{s}}\right)\;d\theta ,$$

and where the function *h* does not have a explicit form but it is the solution to a differential equation. Specifically, *h*(*θ*) = *g*(*θ*)/sin (*θ*) where *g* is the unique solution (for the exact functional space in which this unique solution is defined, the reader is referred to [5, Lemma 2.3])

$$\frac{\nu }{d_{s}}\sin\theta \frac{dg}{d\theta }+\frac{d^{2}g}{d\theta^{2}}=\sin\theta .$$

For an explanation on the meaning of these equations, the reader is referred to [22]. We just point out here that the system (2.6) for $\left(\rho_{g},\Omega_{g}\right)$ corresponds to the so-called self-organized hydrodynamics with repulsion (SOHR) [5] in the case where $\nabla_{x}{\bar{\rho }}_{f}=0$ (i.e. when there is no influence from the obstacles). The SOHR is the continuum version of the Vicsek model with agent-agent repulsion [5].

**Remark 2.1 (Approximation for *ρ*_*f*_ and blow-up).**

The density *ρ*_*f*_ may take negative values: in that case the continuum simulations will be stopped. Note also that solutions may ‘blow-up’ in the sense that particle densities may concentrate at points in space. This happens essentially when the dynamics leads to very concentrated particle clusters, leading to a highly negative Laplacian that decreases drastically the local value of the obstacle density given by equation (2.7). We stress the fact that this is not due to the choice of the numerical parameters but it is intrinsically contained in the macroscopic dynamics. Indeed, nothing prevents the macroscopic model from generating very concentrated solutions. We will show the solutions of the macroscopic model before blow up for the sake of illustration and show that we still observe a very good correspondence with the microscopic dynamics even in these extreme regimes.

## 3. Patterns: phase diagrams

### 3.1. Discrete dynamics

#### 3.1.1. Simulation setup

We here show some simulations of the discrete model (2.1) to give an overview of the different types of patterns that emerge depending on the values of the parameters. Simulations are performed with *N* = *M* = 3000 agents and obstacles initially distributed uniformly in the periodic domain *U* = [0, 1] × [0, 1]. We also suppose that anchor points *Y*_*k*_ for the obstacles are uniformly distributed in *U*, and fix the initial agent direction to *π*/4.

We consider the following expressions for the agent-agent and agent-obstacle repulsion potentials:

3.1 $$\psi (x)=\frac{6\mu }{\pi r_{R}^{2}}{\left(1-\frac{\left|x\right|}{r_{R}}\right)}_{+}^{2}\;\mathrm{and}\;\phi (x)=\frac{3C_{\phi }}{2\pi \tau }{\left(1-\frac{\left|x\right|}{\tau }\right)}_{+}^{2},$$

where

$$x_{+}^{2}=\left\{ \begin{array}{cc} x & \text{if }x^{2}\in R_{+},\\ 0 & \text{if }x<0. \end{array}\right.$$

Therefore, both potentials are compactly supported and act in a radius *r*_*R*_ > 0 for agent-agent repulsion and a radius *τ* > 0 for agent-obstacle repulsion. Note that the constants have been chosen such that

$$\mu =\int \psi (x)\;dx\;\text{and}\;C_{\phi }=\int |\nabla \phi |(x)\;dx.$$

We fix a set of parameters as described in table 1, and focus our study on the interplay between three parameters: the obstacle spring stiffness *κ*, the agent friction *ζ* and the agent-agent repulsion intensity *μ*.

**Table 1.** Parameters used for the discrete simulations of figure 2. The various values considered for *μ*, *ζ*, *κ* are specified in the caption of figure 2.

parameters	value	description
*N*	3000	number of obstacles
*M*	3000	number of agents
*u*_0_	1	agent speed
*r*_*R*_	0.075	agent-agent repulsion distance
*r*_*A*_	0.1	agent-agent alignment distance
*ν*	2	agent-agent alignment intensity
*τ*	0.15	agent-obstacle repulsion distance
$C_{\phi }$	5	agent-obstacle repulsion intensity
*d*_*s*_	0.02	noise in the agents’ orientation
*η*	1	obstacle friction
*d*_0_	0	obstacle positional noise
*μ*	various	agent-agent repulsion intensity
*ζ*	various	friction constant of the agents
*κ*	various	spring constant coefficient

#### 3.1.2. Phase diagram

**Figure 2.**
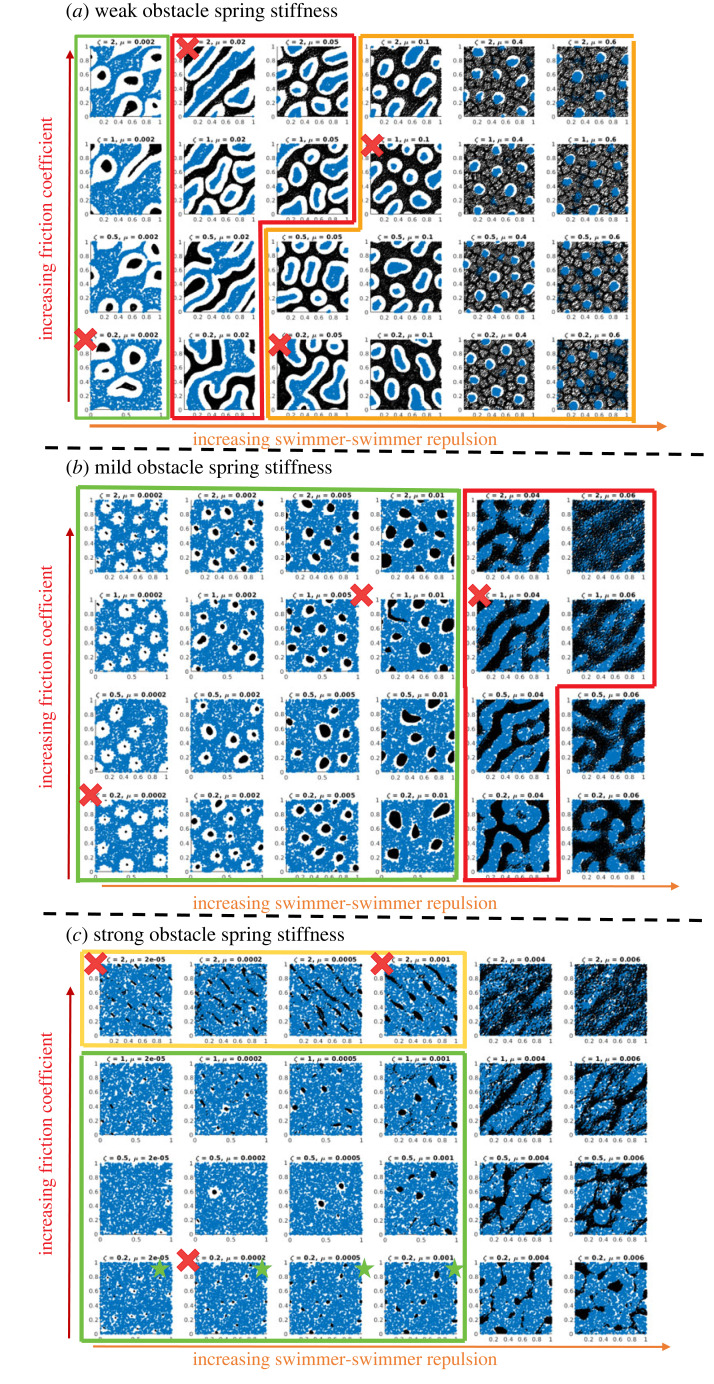
Simulations of the discrete model for the parameters indicated in table 1. Agents are represented as black arrows giving their direction of motion, obstacles are represented as blue circles. (*a*) For weak obstacle stiffness *κ* = 10, (*b*) for mild obstacle stiffness *κ* = 100 and (*c*) for large obstacle stiffness *κ* = 1000. In each panel, the vertical axis represents different values of the friction coefficient *ζ* (from bottom to top: *ζ* = 0.2, 0.5, 1, 2 for (*a*,*b*) and *ζ* = 0.2, 1, 2, 5 for (*c*)); and the horizontal axis represents different values of the agent-agent repulsion *μ*: (*a*) *μ* ∈ {0.002, 0.02, 0.05, 0.1, 0.4, 0.6}, (*b*) *μ* ∈ {0.0002, 0.002, 0.005, 0.01, 0.04, 0.06} and (*c*): *μ* ∈ {2 × 10^−5^, 2 × 10^−4^, 6 × 10^−4^, 2 × 10^−3^, 4 × 10^−3^, 6 × 10^−3^}.

Figure 2 shows the output of the simulations at time *t* = 10: at this time agents and obstacles patterns seem to have reached a steady state. In this figure, agents’ positions and their orientations are represented by black arrows and obstacles' positions with blue dots. The output of the simulations is grouped into three panels: panel (*a*) corresponds to weak obstacle spring stiffness *κ* = 10, and panels (*b*,*c*) correspond to mild *κ* = 100 and strong *κ* = 1000 obstacle spring stiffness, respectively. Inside each panel, we arrange the simulations in a table: right-to-left columns correspond to increasing values of the agent-agent repulsion force *μ*, bottom-to-top rows correspond to increasing values of the friction coefficient *ζ*. Note that the value for the agent-agent repulsion force *μ* is not taken the same in all panels. Indeed, the values for *μ* selected are the ones that make different patterns appear in the simulations. We will justify further the particular choice of the parameters after the linear stability analysis of the continuum equations. Note that the values for *ζ* are also different in (*c*). We refer the reader to the caption of figure 2 for the exact choices for the parameter values of *μ* and *ζ*. Finally, we point out that the figures marked with a red cross are the ones for which the videos can be found in the electronic supplementary material, appendix A for more details.

From figure 2, we observe that a rich variety of agents’ patterns emerges when varying the spring stiffness *κ*, the intensity of the agent-agent repulsion *μ*, and the friction coefficient *ζ*.

We classify these patterns into four main types and we outline the parameter regions corresponding to each with frames of different colours in figure 2:

— trails of agents (framed in red): agents organize into trails inside the obstacle pool. This behaviour is mainly observed for weak and mild obstacle spring stiffness (*κ* = 10 (*a*) and *κ* = 100 (*b*) of figure 2, respectively);
— honeycomb organization of the agents (framed in orange): for small obstacle spring stiffness *κ* = 10 (figure 2*a*) and mild agent-agent repulsion *μ* > 0.1 (middle columns), we observe that the agents organize into fixed honeycomb structures, framing the obstacles which concentrate into aggregates of different sizes and shapes (not necessarily round). We point out that this pattern was not detected in the previous publication [22];
— travelling bands of agents (framed in yellow): only observed for large values of the obstacle spring stiffness *κ* = 10^3^ and large agent friction with the environment *ζ* = 5, here the agents organize into bands perpendicular to their direction of motion. The width of the bands increases with the agent-agent repulsion intensity *μ* (from left to right plots of the first row of panel (*c*)); and
— clusters of agents (framed in green): agents organize into clusters more or less round depending on the regime of parameters. Cluster formation appears in all regimes of obstacle spring stiffness *κ* = 10, 10^2^, 10^3^ (all three panels), and the size of the clusters changes depending on the obstacle spring stiffness *κ* and on the agent-agent repulsion intensity *μ* but seems independent of the agent friction *ζ*. Particularly, we observe that the cluster sizes increase with *μ*, until a point is reached in which *μ* is so large that agent-agent repulsion counteracts all the other aggregation forces (right columns of figure 2). Moreover, the parameter *μ* acts as a phase transition parameter between different types of patterns. During the transition from clustered to near-homogeneous agent distributions with increasing repulsion intensity *μ*, we observe a passage to other pattern types such as trails (for weak *κ* = 10 or mild *κ* = 100 obstacle stiffness), or honeycomb organizations (for weak obstacle stiffness). Finally, we note that for large obstacle spring stiffness *κ* and small agent friction *ζ* (bottom row of panel (*c*), simulations marked with a green star) we observe the formation of 'pinned’ clusters where the agents are grouped into very small clusters that do not move (see electronic supplementary material, appendix A for access to the videos)

Each of these agent patterns is surrounded by obstacles that are kept at a given distance from the agents. This distance depends on the stiffness of the obstacles’ springs *κ* and the agent-agent repulsion intensity *μ*. On the one hand, if obstacles are loose enough (i.e. *κ* is small), the repulsion force between the agents and the obstacles may be large enough to keep them both at approximately the obstacle-agent repulsion distance *τ* (defined in the potential *ϕ*, equation (3.1)). On the other hand, agent-agent repulsion opposes this effect, by giving the agent population force to go against the pressure exerted by the obstacle pool. We indeed observe that increasing the agent-agent repulsion force *μ* (left-to-right columns of figure 2) decreases the typical distance between the agent structures and the obstacles.

**Remark 3.1.**

It is noteworthy that the agent-based model features stochastic terms through the noise in position and orientation, as well as in the choice of the initial condition. Note that we set the noise in position to *d*_0_ = 0 in all our simulations, but the noise in orientation is fundamental since the macroscopic model is obtained in the limit of large noise (and large alignment rate, see the scaling assumptions in §2.2.1). As previously shown on the Vicseck model, the noise has a structural role in the formation of patterns, as it enables disorder to be controlled. In [32], for instance, the authors study the phase transitions for kinetic models describing self-propelled particles interacting through alignment, and highlight how the transitions between isotropic and non-isotropic equilibria are controlled by the competition between alignment and noise. They show in particular that the ratio between alignment and noise entirely determines the phase transition features (number and nature of equilibria, stability, convergence rate and hysteresis), therefore documenting exhaustively the role of noise in these types of systems.

### 3.2. Continuum dynamics

In this section, we show numerical simulations of the continuum equations (2.6) using the numerical scheme detailed in electronic supplementary material, appendix C.

#### 3.2.1. Simulation setup

We perform simulations of the continuum model on the periodic domain *U* = [0, 1] × [0, 1] discretized with space step Δ*x* ≈ 6.7 × 10^−3^ (150 discretization points in each direction). The initial homogeneous agent direction $\Omega_{0}$ is set to *π*/4, and initial agent density *ρ*_*g*_ is a small perturbation of a uniform distribution with $\int_{\Omega }\rho_{g}=1$. In order to compare the numerical results with the discrete model, we use the same parameters as for the discrete simulations presented in §3.1 (table 2).

**Table 2.** Parameters used for the simulations of the continuum equations (2.6) shown in figure 3. The constants *d*_1_, *d*_2_, *d*_3_ depend only on *ν*/*d*_*s*_ and are obtained by computing the expressions (2.10) and (2.11).

parameters	value	description
*h*	≈6.7 × 10^−3^	step-size spatial discretization
*v*_0_	1	agent speed
*r*_*A*_	0.15	agent-agent alignment distance
*d*_*s*_/*ν*	0.01	parameter coming from alignment forces
*τ*	0.15	agent-obstacle repulsion distance
$C_{\phi }$	5	agent-obstacle repulsion intensity
*η*	1	obstacle friction
*γ*_*s*_	28 × 10^−4^	viscosity coefficient
*μ*	various	agent-agent repulsion intensity
*ζ*	various	agent friction constant
*γ*	various	*γ* = *η*/*κ*

Note that the agent-agent alignment distance at the continuum level is chosen to be *r*_*A*_ = 0.15 whereas for the discrete simulations it was 0.1. This choice corresponds to having rescaled *r*_*A*_ approximately by a scaling factor ε=0.5, i.e. $r_{A}^{\prime }=\sqrt{\varepsilon }r_{A}$, where *r*_*A*_′ = 0.1 is the parameter used in the discrete simulation (see the scaling assumption (b) in §2.2.1). Note that only the ratio *d*_*s*_/*ν* is relevant for the continuous model, independently of their individual values. We therefore just ensure that this ratio is kept the same as for the discrete simulations and use *d*_*s*_/*ν* = 0.01.

#### 3.2.2. Phase diagram

**Figure 3.**
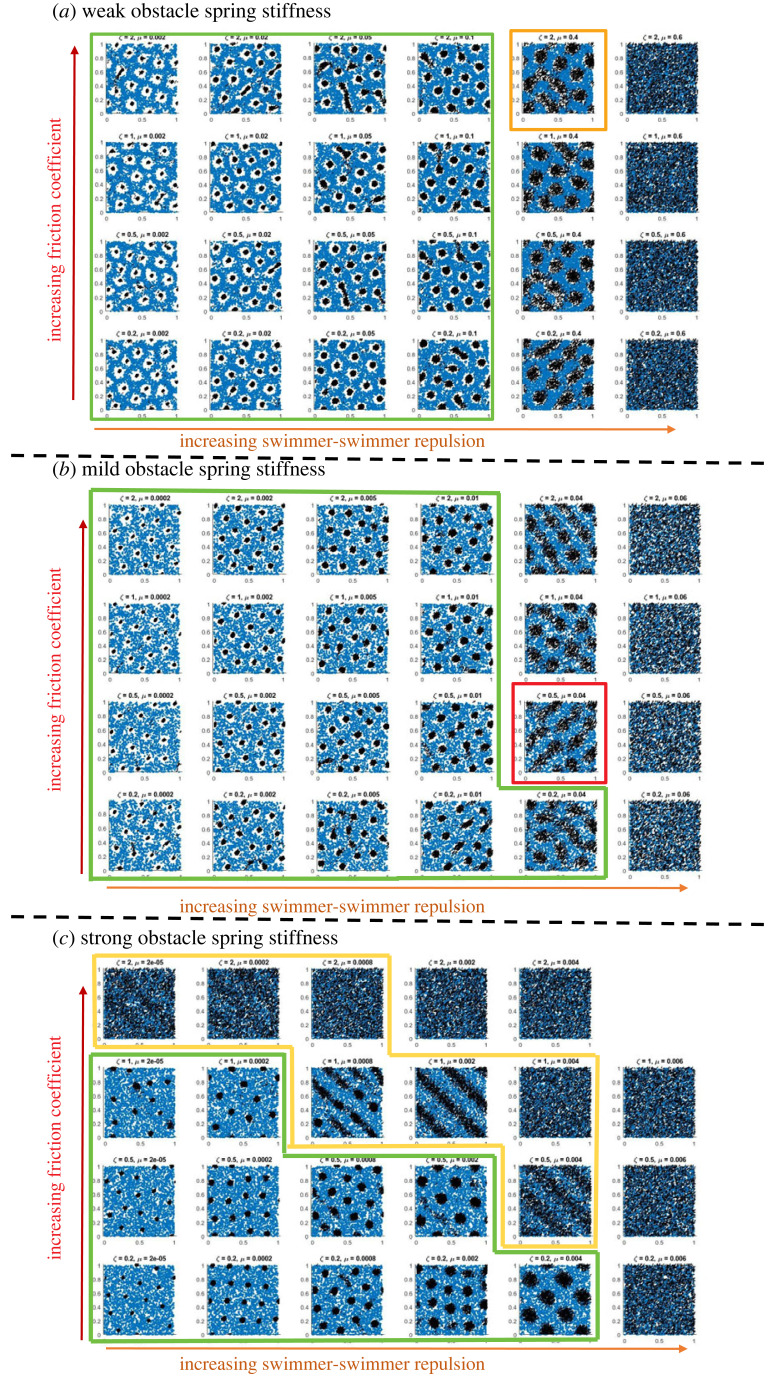
Simulations of the continuum model (2.6) for the parameters indicated in table 2. Agents (randomly distributed from the distribution *ρ*_*g*_(*x*, *t*)) are represented as black arrows of orientation *π*/4, obstacles (randomly distributed from the distribution *ρ*_*f*_(*x*, *t*)) are represented as blue circles. (*a*) For weak obstacle stiffness *κ* = 10, (*b*) for mild obstacle stiffness *κ* = 100, (*c*) for large obstacle stiffness *κ* = 1000. In each panel, the vertical axis represents different values of the friction coefficient *ζ* (from bottom to top: *ζ* = 0.2, 0.5, 1, 2 and the horizontal axis represents different values of the agent-agent repulsion *μ*): (*a*) *μ* ∈ {0.002, 0.02, 0.05, 0.1, 0.4, 0.6}, (*b*) left column: *μ* ∈ {0.0002, 0.002, 0.005, 0.01, 0.04, 0.06} and (*c*) *μ* ∈ {2 × 10^−5^, 2 × 10^−4^, 6 × 10^−4^, 2 × 10^−3^, 4 × 10^−3^, 6 × 10^−3^}.

We present the output of the continuum simulations. To facilitate the comparison with the discrete system, we adopt the same representation as the one presented in figure 2. In particular, the continuum densities are discretized as follows: at a simulation time *t* we distribute randomly *N* = 3000 agent points in the domain according to the distribution *ρ*_*g*_( · , *t*), and similarly for the obstacle points using *ρ*_*f*_( · , *t*). In figure 3, we show the simulation results at the final time of the simulation, corresponding either to the time before blow-up or appearance of negative density for the obstacles (see remark 2.1) or to *t* = 10, as for the discrete simulations. As in figure 2, the simulations are separated in three panels: panel (*a*) is obtained for weak obstacle stiffness *κ* = 10, and panels (*b*) and (*c*) are for *κ* = 100 and *κ* = 1000, respectively. In each panel, we organize the simulations in tables for which bottom-to-top rows correspond to increasing values of the friction coefficient *ζ*, while left-to-right columns correspond to increasing values of the agent-agent repulsion force intensity *μ*. See the legend of figure 3 for more details on the parameter values considered for *ζ* and *μ*.

In figure 3, we observe different patterns for the agents, each framed using the same colour code as for the discrete simulations: cluster formation (framed in green, present in all three panels), travelling bands (framed in yellow, (*c*)), trails (framed in red, (*b*)), near-honeycomb structures (framed in orange, (*a*)), uniform distributions (unframed) and in-between states. Here again, increasing the obstacle spring stiffness *κ* (from top to bottom panels) decreases the distance between agents and obstacles (i.e. the white area around the agents is reduced with increasing *κ*). We also observe that increasing the agent-agent repulsion intensity *μ* increases the size of the agent clusters and this parameter again serves as a transition parameter between clusters and uniform distribution of the agents, passing through honeycomb structures (first row of panel (*a*)), trails (third row of panel (*b*)) or travelling bands (first three rows of panel (*c*)). The effect of the friction parameter *ζ* becomes more relevant for large values of *κ*. For example, in (*c*), the parameter *ζ* serves as a transition parameter between clusters, trails and uniform states.

**Comparing phase diagrams.** We compare the two phase diagrams from the discrete simulations in figure 2 and the continuum simulations in figure 3. Note, though, that there is not an exact correspondence of the values for the parameter *ζ* used in (*c*) for the two cases.

It is noteworthy that the patterns observed with the continuum simulations are similar to the patterns of the discrete simulations (figure 2) for strong and mild obstacle spring stiffness (compare figures 3*b*,*c* and 2), while the two models lead to different types of behaviour in the weak obstacle stiffness regime (*a*). These are expected results since the continuum model has been obtained in a strong obstacle spring stiffness regime (1/*κ* ≈ 0). As a result, the continuum model seems to be unable to produce the rich variety of patterns offered by the discrete model when considering loose obstacles. Also, we do not observe the pinned state with the continuum model, which appeared with the discrete dynamics when considering large obstacle spring stiffness *κ* and small agent friction *ζ*. Even though pinned-states are observed for large values of *κ*, they correspond to states where agents collapse into a very small cluster and then the numerical simulations of the continuum equations blow-up due to a high concentration of the agent density *ρ*_*g*_ (see remark 2.1).

## 4. Linear stability of uniform states

### 4.1. Analysis of the continuum model

Continuum equations are amenable to linear stability analysis around constant solutions or uniform states. This is useful because the presence of instabilities signals the formation of patterns. In this section, we obtain an explicit condition for the stability of uniform states.

Before stating the main result, we introduce the following notation: denote by $\hat{\phi }$ the Fourier transform of *ϕ* defined as, for $k\in R^{2}$

$${\hat{\phi }}_{k}\;:={\hat{\phi }}_{\left|k\right|}=\hat{\phi }(k)=\int_{R^{2}}\;e^{-ik\cdot x}\phi (x)\;dx\in R.$$

Note that *ϕ* is assumed rotationally invariant, therefore $\hat{\phi }$ is real and rotationally invariant (so we abused notation and wrote ${\hat{\phi }}_{\left|k\right|}$ instead of ${\hat{\phi }}_{k}$).

**Theorem 4.1 (Linear instability).**

*Consider fixed constant values* *ρ*_0_ > 0 *and* $\Omega_{0}\in S^{1}$. *Then, the linearized system of* (*2.6*) around $\left(\rho_{0},\Omega_{0}\right)$ *is unstable if and only if*

4.1 $$\text{there exists }z>0\text{ such that }z^{2}{\left({\hat{\phi }}_{z}\right)}^{2}>\mu \kappa .$$

The proof of the theorem is given later. First, we derive sufficient conditions for the system to be stable:

**Corollary 4.2 (Conditions for stability).**

*Suppose that* *ϕ* *is absolutely continuous, rotationally invariant, and* *ϕ*, *ϕ*′ ∈ *L*^1^. *Then, it holds that*

4.2 $$c_{0}\;:\;=\underset{z\in R_{+}}{\max}z^{2}{\left({\hat{\phi }}_{z}\right)}^{2}<\infty$$

*and if* *μκ* > *c*_0_, *then the continuum equations* (*2.6*) *are linearly stable*.

*Moreover, if* *ϕ* *is given by* (*3.1*), *define* $c_{0}^{\prime }=c_{0}/C_{\phi }^{2}$. *It holds that the constant* *c*_0_′ *is independent on the obstacle-agent repulsion radius* *τ* *and the intensity* $C_{\phi }$ *and the system is stable whenever*

$$\frac{\mu \kappa }{C_{\phi }^{2}}>c_{0}^{\prime }.$$

**Proof.**

Since by assumption *ϕ* is absolutely continuous and *ϕ*, *ϕ*′ ∈ *L*^1^, we have that $\left|{\hat{\phi }}^{\prime }(k)|\;=\;|k\|\hat{\phi }(k)\right|$. Moreover, since *ϕ*′ ∈ *L*^1^, then ${\hat{\phi }}^{\prime }$ is bounded. Therefore, ${\left|k\right|}^{2}|\hat{\phi }(k){|}^{2}$ is bounded and *c*_0_ is finite. In this case, for *μκ* > *c*_0_ the instability condition (4.1) does not hold, so the system is stable.

In the particular case where *ϕ* takes the shape given in (3.1), one can check that the following self-similarity condition holds:

$$|k|{\hat{\phi }}_{k}=\tau |k|{\hat{\phi }}^{\left(1\right)}(\tau |k|)\;\forall \tau ,$$

where *ϕ*^(1)^ corresponds to *ϕ* when taking *τ* = 1. Therefore, it holds that

$$C_{\phi }^{2}c_{0}=\max_{k}{\left|k\right|}^{2}{\left({\hat{\phi }}_{k}\right)}^{2}=\max_{k}{\left(\tau |k|\right)}^{2}{\left({\hat{\phi }}^{\left(1\right)}(\tau |k|)\right)}^{2}=\max_{y}|y{|}^{2}{\left({\hat{\phi }}^{\left(1\right)}(|y|)\right)}^{2},$$

and so *c*_0_ is independent of *τ*. The rest of the corollary follows: the value of *c*_0_′ is also clearly independent of $C_{\phi }$ as it is just a multiplicative factor of *ϕ*. ▪

**Remark 4.3 (Limiting case of pillar obstacles).**

In the case where the obstacles are fixed pillars, i.e. the case where *κ* → ∞, then the uniform distribution of agents and pillars is always a stable solution. The effect of this limiting case is that the equations for the agents on $\left(\rho_{g},\Omega \right)$ become decoupled from the obstacles’ density *ρ*_*f*_ = *ρ*_*A*_, which is just constant (take the formal limit *κ* → ∞ on the continuum equations (2.6)). Therefore, there is a striking behavioural change between static obstacles and obstacles that can move a bit (anchored at a fixed point via a very stiff spring). This shows that, in this particular setup, the fact that the agents are able to modify their environment is crucial for interesting patterns to emerge.

**The role of the parameters.** From the instability condition (4.1), we observe that the main drivers of the formation of instabilities are: the shape of the agent-obstacle repulsion potential *ϕ*, the obstacle-spring stiffness *κ*, and the agent-agent repulsion intensity *μ*. High agent-agent repulsion—high values of *μ*—has a stabilizing effect while high agent-obstacle repulsion—high values of $C_{\phi }$—has a destabilizing effect, and vice versa. Also, high values of the spring constant *κ* have a stabilizing effect and small values have the opposite effect.

From corollary 4.2, in the case when *ϕ* is given in (3.1) the ratio given by

4.3 $$b_{p}\;:\;=\frac{\mu \kappa }{C_{\phi }^{2}c_{0}^{\prime }}$$

is the single value that acts as a bifurcation parameter. However, the obstacle-agent repulsion radius *τ* plays a role in determining the size of the patterns (figure 3). Also, from the instability condition (4.1) and corollary 4.2, for typical shapes of the potential *ϕ*, we expect to have stability for small and large values of the wavevector *k* but instabilities can appear at intermediate values whenever *c*_0_ > *μκ* (where *c*_0_ is given in (4.2)).

The rest of this section is devoted to the proof of theorem 4.1.

**Proof of theorem 4.1.**

We start by linearizing the continuum equations (2.6) around $\left(\rho_{0},\Omega_{0}\right)$ by expanding the solution using a small perturbation parameter *β*

4.4 $$\rho_{g}=\rho_{0}+\beta \rho_{1}+O(\beta^{2}),\;\Omega =\Omega_{0}+\beta \Omega_{1}+O(\beta^{2})\;\mathrm{and}\;|\Omega |=1.$$

Dropping the higher order terms, we obtain the linearized system (where the over-script bar notation is defined in equation (2.8))

4.5a $$\partial_{t}\rho_{1}+d_{1}\Omega_{0}\cdot \nabla \rho_{1}+d_{1}\rho_{0}\nabla \cdot \Omega_{1}=\bar{\mu }\rho_{0}\Delta \rho_{1}+\rho_{0}\bar{\lambda }(\Delta^{2}{\bar{\bar{\rho }}}_{1}-\gamma \Delta^{2}\partial_{t}{\bar{\bar{\rho }}}_{1}),$$

4.5b $$\rho_{0}\partial_{t}\Omega_{1}+\rho_{0}d_{2}(\Omega_{0}\cdot \nabla )\Omega_{1}+d_{3}P_{\Omega_{0}^{⊥}}\nabla \rho_{1}=\gamma_{s}\rho_{0}P_{\Omega_{0}^{⊥}}\Delta \Omega_{1}$$

4.5c $$\mathrm{and}\;\Omega_{0}\cdot \Omega_{1}=0,$$

where Δ^2^ is the bi-Laplacian, i.e. Δ^2^*ρ* = Δ(Δ*ρ*), and $P_{\Omega_{0}^{⊥}}$ is the orthogonal projection on $\Omega_{0}^{⊥}$. Note also that $\bar{\mu }=\mu /\zeta$, *γ* = *η*/*κ* and $\bar{\lambda }=\rho_{A}/(\kappa \zeta )$ (we assume *ρ*_*A*_ = 1).

We now define the functions $F,G\;:\;R_{+}\to R$ by

4.6 $$\begin{array}{cc} & F(z)\;:=z^{2}\frac{\rho_{0}}{\zeta }\left(\frac{1}{\kappa }z^{2}{\left({\hat{\phi }}_{z}\right)}^{2}-\mu \right)\\ \mathrm{and}\; & G(z)\;:=1+\rho_{0}\frac{\eta }{\kappa^{2}\zeta }z^{4}{\left({\hat{\phi }}_{z}\right)}^{2}>0, \end{array}\}$$

and given *k* ∈ *R*^2^, we denote by *k*_0_, *k*_1_ the quantities

4.7 $$k_{0}=(k\cdot \Omega_{0})\;\mathrm{and}\;k_{1}=(k\cdot \Omega_{0}^{⊥}),$$

where $\Omega_{0}^{⊥}$ is the image of $\Omega_{0}$ by the rotation of angle *π*/2. Theorem 4.1 is then a direct consequence of the following proposition.

**Proposition 4.4.**

*System* (*4.5*) *allows for non-trivial plane wave solutions, i.e. solutions of the form*

4.8 $$\rho_{1}(x,t)=\tilde{\rho }\;e^{ik\cdot x+\alpha t}\;\mathrm{and}\;\Omega_{1}(x,t)=\tilde{\;\Omega }\;e^{ik\cdot x+\alpha t},$$

*where* $k\in R^{2}$ *is the wavevector*, α∈C, $\tilde{\rho }\in C$ *and* $\tilde{\;\Omega }\in C^{2}$, *and* $\left(\tilde{\rho },\tilde{\Omega })\neq (0,0\right)$ *if and only if* *α* *and* *k* *fulfil the following dispersion relations*:

***Case A:*** $k∥\Omega_{0}$

*Option 1:* $\tilde{\rho }\neq 0$, $\tilde{\;\Omega }=0$,

4.9 $$\alpha =\alpha_{1}(k)\;:\;=-i\frac{d_{1}k_{0}}{G(|k_{0}|)}+\frac{F(|k_{0}|)}{G(|k_{0}|)}.$$

*Option 2:* $\tilde{\rho }=0$, $\tilde{\;\Omega }\neq 0$.

4.10 $$\alpha =\alpha_{2}(k)\;:\;=-id_{2}k_{0}-{\left|k\right|}^{2}\gamma_{s}.$$

***Case B:*** $k∦\Omega_{0}$. *Then*, *α* *is a root of the following polynomial of degree 2:*

4.11 $$\begin{array}{rl} & \alpha^{2}G+\alpha [G{\left|k\right|}^{2}\gamma_{s}-F+ik_{0}(Gd_{2}+d_{1})]\\ & \;+d_{1}(\rho_{0}d_{3}k_{1}^{2}-d_{2}k_{0}^{2})-{\left|k\right|}^{2}\gamma_{s}F+i(d_{1}k_{0}{\left|k\right|}^{2}\gamma_{s}-d_{2}k_{0}F)=0. \end{array}$$

*The real parts of* *α* *are negative if and only if the following holds:*

4.12 $$G(k){\left|k\right|}^{2}\gamma_{s}-F(k)>0,$$

*and*

4.13 $$H(k)\;:={\left[G(k){\left|k\right|}^{2}\gamma_{s}-F(k)\right]}^{2}d_{1}d_{3}k_{1}^{2}$$

4.14 $$\;-\gamma_{s}F(k){\left|k\right|}^{2}[{\left(d_{1}-d_{2}G(k)\right)}^{2}k_{0}^{2}+{\left[G(k){\left|k\right|}^{2}\gamma_{s}-F(k)\right]}^{2}]>0.$$

**Proof of proposition 4.4.**

Substituting the plane wave ansatz into the equation yields

4.15a $$\tilde{\rho }\alpha +i\tilde{\rho }d_{1}(\Omega_{0}\cdot k)+i\rho_{0}d_{1}(\tilde{\;\Omega }\cdot k)=-{\left|k\right|}^{2}\bar{\mu }\rho_{0}\tilde{\rho }+{\left|k\right|}^{4}\bar{\lambda }\rho_{0}\tilde{\rho }{\left({\hat{\phi }}_{k}\right)}^{2}(1-\gamma \alpha ),$$

4.15b $$\rho_{0}\alpha \tilde{\;\Omega }+i\rho_{0}d_{2}\tilde{\;\Omega }(\Omega_{0}\cdot k)+i\tilde{\rho }d_{3}P_{\Omega_{0}^{⊥}}k=-{\left|k\right|}^{2}\rho_{0}\gamma_{s}\tilde{\;\Omega }$$

4.15c $$\mathrm{and}\;\Omega_{0}\cdot \tilde{\;\Omega }=0,$$

or (if $\tilde{\Omega }=\omega \Omega_{0}^{⊥}$)

$$(G(|k|)\alpha -F(|k|)+id_{1}k_{0})\tilde{\rho }+i\rho_{0}d_{1}k_{1}\omega =0$$

and

$$id_{3}k_{1}\tilde{\rho }+\rho_{0}(\alpha +id_{2}k_{0}+{\left|k\right|}^{2}\gamma_{s})\omega =0.$$

This is a homogeneous linear system in $\left(\tilde{\rho },\omega \right)$ which has a non-trivial solution if and only if the determinant of the system is 0, i.e.

4.16 $$(G(|k|)\alpha -F(|k|)+id_{1}k_{0})(\alpha +id_{2}k_{0}+{\left|k\right|}^{2}\gamma_{s})+d_{1}d_{3}k_{1}^{2}=0.$$

If *k*_1_ = 0, there are two roots corresponding to either bracket being zero. This leads to (4.9) or (4.10). If *k*_1_ ≠ 0, we can recast (4.16) in (4.11).

To determine the sign of the real part of *α*, we use the Routh–Hurwitz criterion for polynomials with complex coefficients [33,34]. In our case, the Routh–Hurwitz criterion states that the Re(*α*) < 0 for all solutions *α* if and only if expressions (4.12) and (4.13) hold. ▪

With proposition 4.4, we conclude the proof of theorem 4.1 as follows. Suppose (4.1) holds and let *z*_0_ > 0 be such that $z_{0}^{2}{\hat{\phi }}_{z_{0}}^{2}>\mu \kappa$. Let $k=z_{0}\Omega_{0}$. Then *k*_0_ = *z*_0_ and *k*_1_ = 0. So *F*(|*k*|) = *F*(*z*_0_) > 0 and *α* = *α*_1_(*k*) is such that Re(*α*) > 0. Hence, the linearized system is unstable.

Suppose now (4.1) does not hold, i.e. $z^{2}{\hat{\phi }}_{z}^{2}<\mu \kappa$, for all $z\in R_{+}$. Then, *F*(|*k*|) < 0, for all $k\in R^{2}$. It results that Re(*α*_1_(|*k*|)) < 0, Re(*α*_2_(|*k*|)) < 0. Furthermore (4.12) and (4.13) are obviously satisfied for all $k\in R^{2}$. Hence the system is stable. ▪

### 4.2. Numerical validation of the linear stability analysis

In this section, we compare the pattern predictions given by the linear stability analysis with the results obtained from numerical simulations. This way we check that the linear stability analysis truly captures pattern formation, i.e. that nonlinear effects are of second order and most of the patterns characteristics are captured by linear effects.

#### 4.2.1. Predictions from the theoretical analysis and qualitative agreement with the macroscopic simulations

We start by giving insights on the size and shape of the expected patterns based on the theoretical predictions offered by the stability analysis. To this end, we consider perturbations introduced in the stability analysis (see proposition 4.4), around the homogeneous density *ρ*_0_ = 1 and in constant direction $\Omega_{0}\in S^{1}$. As we are particularly interested in characterizing the patterns corresponding to clusters or bands, we will focus on the theoretical values for wave vectors parallel to $\Omega_{0}$ and parallel to $\Omega_{0}^{⊥}$:

$$k_{∥}^{\mathrm{th}}=\underset{k∥\Omega_{0}}{\text{argmax Re}}(\tilde{\alpha }(k))\;\mathrm{and}\;k_{⊥}^{\mathrm{th}}=\underset{k∥\Omega_{0}^{⊥}}{\text{argmax Re}}(\alpha (k)),$$

where $\tilde{\alpha }(k)$ corresponds to case A (equation (4.9)), *α*(*k*) corresponds to case B (larger root of equation (4.11), computed numerically) and the symbol ‘Re’ indicates the real part. With these wavevectors maximizing the real part of *α*, we define the quantities

$$S_{1}^{\mathrm{th}}=\frac{2\pi }{\left|k_{∥}^{\mathrm{th}}\right|}\;\mathrm{and}\;S_{2}^{\mathrm{th}}=\frac{2\pi }{\left|k_{⊥}^{\mathrm{th}}\right|}.$$

These quantities give the size of the expected patterns in each direction. We will also compute the maximal growth rates of the perturbations in these two directions

$$\alpha_{\max}^{∥}=\underset{k∥\Omega_{0}}{\text{max Re}}(\tilde{\alpha }(k))\;\mathrm{and}\;\alpha_{\max}^{⊥}=\underset{k∥\Omega_{0}^{⊥}}{\text{argmax Re}}(\alpha (k)).$$

Equipped with these quantifiers, we now study the influence of the model parameters on the expected pattern shapes and sizes. As predicted by the stability analysis, patterns can be expected if the bifurcation parameter *b*_*p*_ (equation (4.3)) is below 1. We fix $C_{\phi }=5$ and use *ϕ* as in equation (3.1) giving *c*_0_ ≈ 5.6 independent of *τ* as shown in the proof of corollary 1 (definition of *c*_0_ in equation (4.2)). We vary *b*_*p*_ by changing the values of the agent-agent repulsion intensity *μ* and aim to study the influence of the friction constant *ζ*, the obstacle spring stiffness *κ* and the agent-obstacle repulsion distance *τ*. For each subsection, we compare qualitatively these predictions based on the linear stability analysis with simulations of the macroscopic model presented in figure 3.

**Figure 4.**
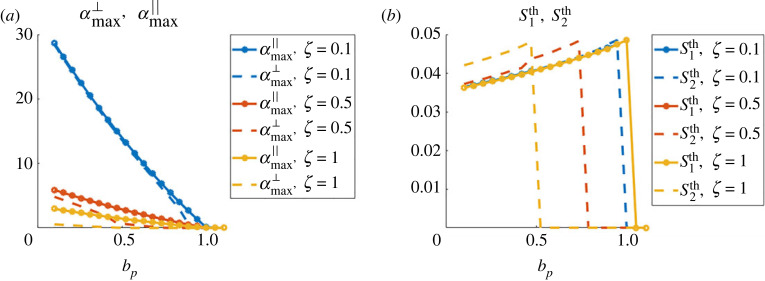
Prediction of the linear stability analysis. (*a*) Values of the maximal growth rate of the plane wave perturbations in the direction of $\Omega_{0}$ (continuous lines) and in the orthogonal direction $\Omega_{0}^{⊥}$ (dashed lines) as functions of the bifurcation parameter *b*_*p*_, for different values of the agent friction *ζ*: *ζ* = 0.1 (blue curves), *ζ* = 0.5 (red curves), *ζ* = 1 (yellow curves). (*b*) Same representation for the size of the perturbations in the two directions $S_{1}^{\mathrm{th}}$ and $S_{2}^{\mathrm{th}}$.

**Influence of the friction constant *ζ*.** First, we fix *κ* = 1000 and *τ* = 0.15, and show in figure 4 the values of $\alpha_{\max}^{∥}$ and $\alpha_{\max}^{⊥}$ (left panel) and of $S_{1}^{\mathrm{th}}$ and $S_{2}^{\mathrm{th}}$ (right panel), as functions of the bifurcation parameter *b*_*p*_ and for different values of the agent friction constant *ζ*: *ζ* = 0.1 (blue curves), *ζ* = 0.5 (red curves), *ζ* = 1 (yellow curves). One can first observe in figure 4*a* that we indeed recover the critical value 1 of the bifurcation parameter, below which perturbations grow (Re(*α*) > 0) and after which they are damped, independently of the value of *ζ*. This shows that *b*_*p*_ is indeed a relevant bifurcation parameter. Moreover, one can observe that perturbations grow faster for smaller values of the friction constant *ζ* (compare the blue and red curves in *a*). From figure 4*b*, we note first that the size of the clusters increases when increasing the bifurcation parameter (here, by increasing the agent-agent repulsion *μ*). These are expected results as stronger agent repulsion leads to higher pressure in the agent population, leading to larger clusters. Secondly, we observe that the size of the patterns is independent on the friction constant *ζ*, but the parameter zone in which patterns are of travelling band type (i.e. $S_{1}^{\mathrm{th}}>0$ and $S_{2}^{\mathrm{th}}=0$) is larger for larger values of *ζ* -compare the yellow and blue dashed curves in *b*. Thus, high friction substrates seem to favour the formation of travelling bands compared to low friction environments, provided the bifurcation parameter is large enough (large obstacle spring stiffness and/or large agent-agent repulsion compared to agent-obstacle repulsion).

**Qualitative comparison with the macroscopic simulations.** The influence of the agent friction *ζ* for *τ* = 0.15 and *κ* = 1000 can be observed in the macroscopic simulations presented in figure 3*c*, comparing the rows together (from bottom to top for increasing values of *ζ*). We first note that in the simulations of the three panels of figure 3, the values considered for the product *μκ* were always the same, i.e.

μκ∈{0.02,0.2,0.5,1,4,6},

and the value of $C_{\phi }=5$ was kept constant, corresponding to the following values for the bifurcation parameter:

$$b_{p}\in \{0.0036,0.0357,0.0893,0.1785,0.7142,1.0713\}.$$

We then observe that in each panel of figure 3, patterns are indeed observed in the first five columns of the tables while the last column displays a homogeneous distribution of agents. This validates the fact that patterns are observed only when the bifurcation parameter *b*_*p*_ is below 1.

Moreover, focusing on the last panel (for which *κ* = 1000), we recover most of the observations predicted by the stability analysis: (*a*) the pattern size increases when increasing the bifurcation parameter (increasing *μ*: compare simulations from left to right in figure 3*c*), (*b*) the zone of parameters showing travelling bands increase when increasing the agent friction *ζ* (compare bottom to top rows of panel (*c*)). Therefore, we obtain a very good qualitative agreement between the simulations of the macro model and the tendencies predicted by the stability analysis as function of *ζ*.

**Figure 5.**
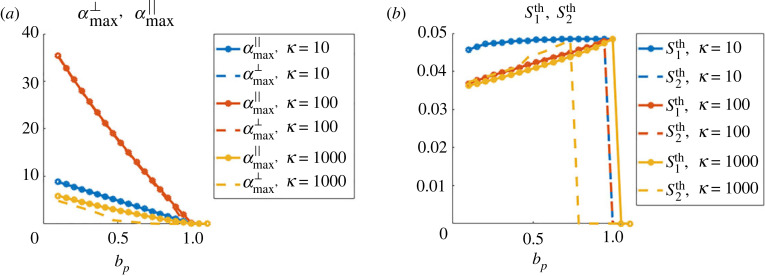
(*a*) Values of the maximal growth rate of the plane wave perturbations in the direction of $\Omega_{0}$ (continuous lines) and in the orthogonal direction $\Omega_{0}^{⊥}$ (dashed lines) as functions of the bifurcation parameter *b*_*p*_, for different values of the obstacle spring stiffness *κ*: *κ* = 10 (blue curves), *κ* = 100 (red curves), *κ* = 1000 (yellow curves). (*b*) Same representation for the size of the perturbations in the two directions $S_{1}^{\mathrm{th}}$ and $S_{2}^{\mathrm{th}}$.

**Influence of the obstacle spring stiffness *κ*.** Here, we adopt the same representation as in the previous paragraph, but fixing the agent friction constant *ζ* = 0.5 and playing on the obstacle spring stiffness *κ* (we keep the agent-obstacle distance *τ* = 0.15). Figure 5 shows the values of $\alpha_{\max}^{∥}$ and $\alpha_{\max}^{⊥}$ (*a*) and of $S_{1}^{\mathrm{th}}$ and $S_{2}^{\mathrm{th}}$ (*b*), as functions of the bifurcation parameter *b*_*p*_ and for *κ* = 10 (blue curves), *κ* = 100 (red curves) and *κ* = 1000 (yellow curves). From figure 5*b*, we can observe a similar evolution of the pattern size playing on the obstacle spring stiffness as when changing the friction constant *ζ*: increasing the obstacle spring stiffness *κ* slightly increases the zone of parameters favouring the formation of bands of agents (compare yellow and red dashed curves in the right panel). One can particularly note (blue curve of figure 5*b*) that environments composed of loose obstacles (*κ* = 10) will only promote agent clusters the size of which is independent of the value of the bifurcation parameter. Finally, we note from figure 5*a* that the growth rate of perturbations does not evolve monotonically with the spring stiffness *κ*: faster perturbations are observed for *κ* = 100 compared to *κ* = 10 or *κ* = 1000 (compare red with blue and yellow curves in *a*).

**Qualitative comparison with the macroscopic simulations.** The influence of the obstacle spring stiffness *κ* for *τ* = 0.15 and *ζ* = 0.5 can be observed in the macroscopic simulations presented in figure 3, comparing the second rows (starting from the bottom) in each panel ((*a*) for *κ* = 10, (*b*) for *κ* = 100 and (*c*) for *κ* = 1000).

Again, we obtain a very good agreement with the theoretical predictions: (a) the pattern sizes increase when increasing the bifurcation parameter (by increasing *μ*: compare simulations from left to right in each panel), (b) the increase in pattern size as function of *μ* seems less important for *κ* = 10 (panel (*a*)) than for larger obstacle spring stiffness (panels (*b*) and (*c*)), and (c) travelling bands are only observed for *κ* = 1000 (panel (*c*)).

**Figure 6.**
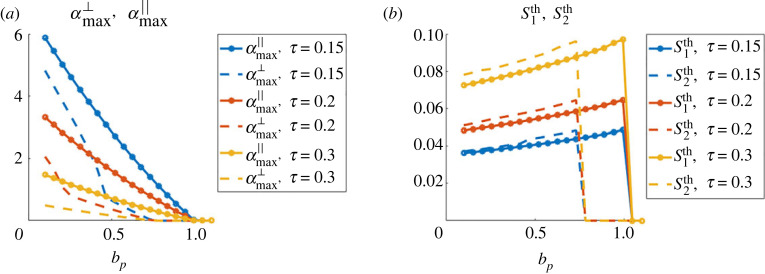
(*a*) Values of the maximal growth rate of the plane wave perturbations in the direction of $\Omega_{0}$ (continuous lines) and in the orthogonal direction $\Omega_{0}^{⊥}$ (dashed lines) as functions of the bifurcation parameter *b*_*p*_, for different values of the agent-obstacle distance *τ*: *τ* = 0.15 (blue curves), *τ* = 0.2 (red curves), *τ* = 0.3 (yellow curves). (*b*) Same representation for the size of the perturbations in the two directions $S_{1}^{\mathrm{th}}$ and $S_{2}^{\mathrm{th}}$.

**Influence of the agent-obstacle repulsion distance *τ***. Finally, we aim to document the role of the agent-obstacle repulsion distance *τ*. We adopt the same methodology as in the two previous paragraphs: we fix *ζ* = 0.5 and *κ* = 1000 and show in figure 6 the values of $\alpha_{\max}^{∥}$ and $\alpha_{\max}^{⊥}$ (*a*) and of $S_{1}^{\mathrm{th}}$ and $S_{2}^{\mathrm{th}}$ (*b*), as functions of the bifurcation parameter *b*_*p*_ and for *τ* = 0.15 (blue curves), *τ* = 0.2 (red curves) and *τ* = 0.3 (yellow curves). We first observe that increasing the value of *τ* slows down the growth of the perturbation modes (compare blue, red and yellow curves of figure 6*a*). Moreover, as predicted by the stability analysis, the critical value of *μ* for which patterns appear does not depend on *τ*: patterns are once again only observed as long as the bifurcation parameter *b*_*p*_ does not exceed the value 1. Secondly, figure 6*b* shows that the agent-obstacle distance *τ* has a strong impact on the size of the clusters: larger *τ* leads to larger agent clusters (compare for instance yellow and blue curves in figure 6*b*), and agent-obstacle repulsion distance does not impact the shape of the patterns (clusters or bands types).

As the simulations of figure 3 have been generated only for *τ* = 0.15, we are not able at this point to compare qualitatively the predictions of the stability analysis with the simulations of the macroscopic model as functions of this parameter. We will however assess the influence of *τ* via a quantitative comparison between the model and the theory in the next section.

Altogether, these results show that agent-agent repulsion favours the spreading of the agents while agent-obstacle repulsion tends to aggregate the agents (and consequently clusters obstacles together). Travelling bands of agents seem to be favoured in low friction environments composed of stiff obstacles, and the size of agent clusters seems to be controlled primarily by the agent-obstacle distance and the bifurcation parameter (ratio between the agent-agent repulsion intensity and the agent-obstacle repulsion intensity).

#### 4.2.2. Quantitative agreement between the macroscopic simulations and the stability analysis

Here, we provide a quantitative assessment of the pattern sizes computed numerically on the simulations of the macroscopic model and the ones predicted by the stability analysis. To this end, we first compute numerically the pattern sizes using the two-dimensional discrete Fourier transform of the agent density at equilibrium $\hat{F}[\rho_{g}]=\hat{F}[\rho_{g}](k)$, and extract the frequency of the two maximal modes $k_{∥},k_{⊥}\in R^{2}$ aligned in the direction of $\Omega_{0}$ and $\Omega_{0}^{⊥}$, respectively

$$k_{∥}=\underset{k∥\Omega_{0}}{\text{argmax}}|\hat{F}[\rho_{g}](k)|\;\mathrm{and}\;k_{⊥}=\underset{k∥\Omega_{0}^{⊥}}{\text{argmax}}|\hat{F}[\rho_{g}](k)|,$$

where | · | is the modulus of a complex number. Then, the theoretical quantifiers $S_{1}^{\mathrm{th}}$ and $S_{2}^{\mathrm{th}}$ will be compared with

$$S_{1}=\frac{2\pi }{\left|k_{∥}\right|}\;\mathrm{and}\;S_{2}=\frac{2\pi }{\left|k_{⊥}\right|}.$$

**Figure 7.**
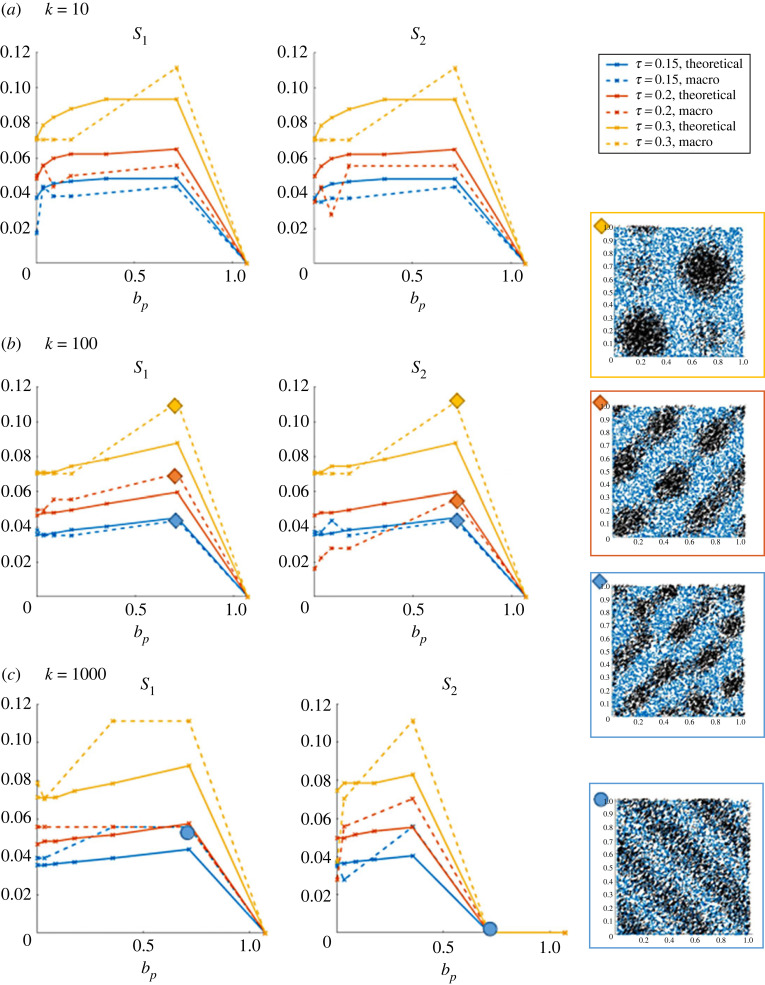
Values of the maximal eigenmodes of the Fourier transform of the numerical solution (dotted curves) and predicted by the stability analysis (plain curves) in direction $\Omega_{0}=(\cos\pi /4,\sin\pi /4)$ (left figures) and $\Omega_{0}^{⊥}$ (right coloured frames). Three different values of the obstacle spring stiffness are considered: *κ* = 10 (*a*), *κ* = 100 (*b*) and *κ* = 1000 (*c*), and three different agent-obstacle repulsion force distances *τ* = 0.15 (blue curves), *τ* = 0.2 (orange curves) and *τ* = 0.3 (yellow curves). Right column: examples of simulations for parameters reported on the graphs: simulations with diamond symbol match (*b*) (yellow frame: clusters; red and blue frame: trails) and the simulation with the circle symbol matches (*c*) (travelling bands).

In figure 7, we show the values of *S*_1_ and *S*_2_ (dotted curves) and $S_{1}^{\mathrm{th}},S_{2}^{\mathrm{th}}$ (plain curves), for three different values of the obstacle spring stiffness *κ* = 10 (*a*), *κ* = 100 (*b*) and *κ* = 1000 (*c*), and three different values of *τ*: *τ* = 0.15 (blue curves), *τ* = 0.2 (orange curves) and *τ* = 0.3 (yellow curves). Note that here *ζ* = 0.5 so that theoretical predictions correspond to figure 6. Simulations are completed for $\Omega_{0}=(\cos\pi /4,\sin\pi /4)$ and *ρ*_0_ = 1.

As one can observe, we obtain a fairly good agreement between the values computed on the numerical solution and the ones predicted by the linear stability analysis as presented in figure 7. As predicted, the size of the repeating patterns increases as *τ* increases (compare blue, red and yellow curves), and as the agent-agent repulsion intensity *μ* increases while staying below the critical threshold μ* (above which the homogeneous steady-state profile is stable), corresponding to *b*_*p*_ = 1. For *κ* = 1000 (figure 7*c*), we also recover the regime of travelling bands predicted for *μ* = 4 × 10^−3^ and here *S*_1_ > 0 and *S*_2_ = 0, i.e. patterns (the travelling bands) are only in the direction $\Omega_{0}$.

## 5. Quantitative assessment of the continuum model

In this section, we aim to compare quantitatively the continuum and discrete models. As our goal is to compare continuous density profiles (continuum model) with clouds of points representing individual positions (discrete model), a method to quantify the ‘proximity’ between these two different types of solutions has to be devised. A first natural choice would be to use the quantifiers defined in the previous section, i.e. to compute the maximal eigenmode of the Fourier transform of the agent distributions from the discrete simulations. This would enable us to construct a space-independent quantifier which could give an insight into the main structures of the discrete model. However, as one can observe in figure 2, the agent and obstacle structures that emerge from the discrete dynamics are not necessarily regularly spaced in the domain, which makes the use of the Fourier transform imprecise for the discrete simulations. Another interesting statistical approach for comparing the models was proposed in [35] and consists in the use of correlation functions that characterize the emergent patterns in the steady state. This approach was successfully used in [36] to study the structural phase transitions in a Vicseck-type model, enabling a new pattern type (namely a ‘cross-sea’ phase) to be detected. However, the analysis of different patterns in this way necessitates the use of proper quantifiers and correlation functions adapted to the shape of the patterns of interest. Due to the richness of the types of motives observed in our case, we opted for the development of a new comparison method, independent of the types of patterns.

In the following section, we propose a new method to compare discrete point clouds and continuum densities which does not require some spatial regularity of the patterns.

### 5.1. Methodology to compare discrete and continuum simulations

In table 3, we summarize the steps of the method we propose to compare discrete and continuum simulations. After generating two simulations (one with the continuum model and one with the discrete dynamics, step 1), we first aim to find the optimal Cartesian mesh on which (i) we interpolate the continuum solution and (ii) we compute the density of the point clouds using a particle-in-cell (PIC) method (step 2, §5.1.1). At the end of this step, both solutions (continuum and discrete) are projected on the same Cartesian mesh. In step 3 (§5.1.2), we then compute a Wasserstein-type distance based on the histograms of the two density distributions.

**Table 3.** Diagram of the methodology used to compare the simulations for the continuum equations (2.6) and the simulation of the discrete dynamics (2.1*a*)–(2.1*b*). PIC, particle-in-cell; EMD, earth movers distance; *W*, Wasserstein distance.

Step 1	simulation of the continuum equations (2.6)	simulation of the particle dynamics (2.1*a*) and (2.1*b*)
	⇓	⇓
Step 2	discretization of the output density on a grid $\Pi_{U}^{\Delta x}$ using the PIC method: $\rho_{\mathrm{mac}}^{\Delta x}$	approximate the particle density on the grip $\Pi_{U}^{\Delta x}$ using the PIC method: $\rho_{\mathrm{mic}}^{\Delta x}$
	→ *compute the optimal grid size Δ*x* using the ℓ^2^ distance (§5.1.1)*	
	↘	↙
Step 3	compare $\rho_{\mathrm{mic}}^{\Delta x}$ and $\rho_{\mathrm{mac}}^{\Delta x}$ with the distance $W(\rho_{\mathrm{mic}}^{\Delta x},\rho_{\mathrm{mac}}^{\Delta x})$	
	→ *computed with the EMD method* (§5.1.2)	

#### 5.1.1. Discretization of the particle density

**Figure 8.**
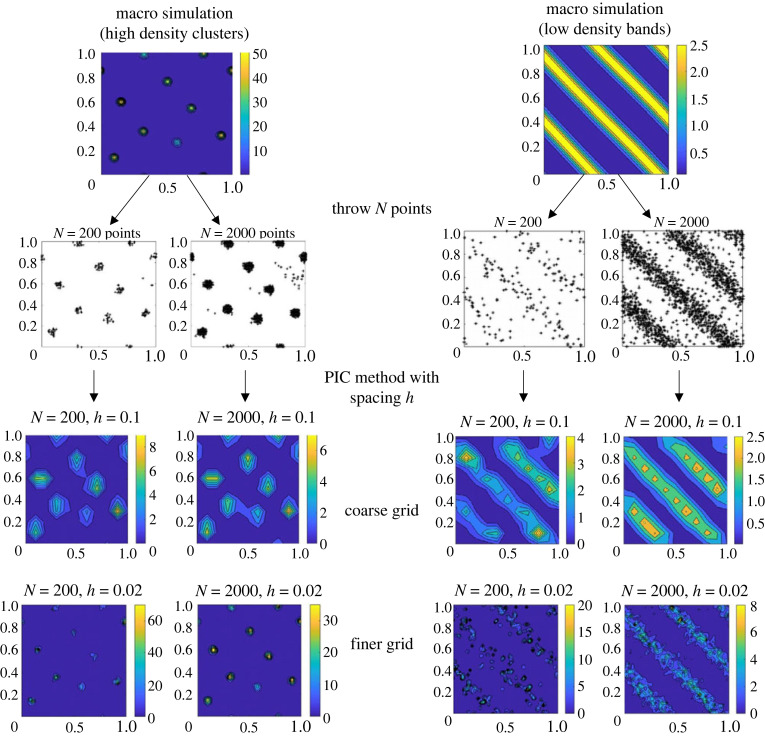
Two examples of the procedure for choosing the optimal grid for the PIC method of the discrete simulations, starting from a continuum simulation with high density aggregates (left column) or low-density bands (right column). The first step (first to second rows) consists in distributing *N* points according to the continuum density distributions (left: for *N* = 200, right for *N* = 2000 points), and the second step (third and fourth rows) computes the approximated density using a PIC method with different spacing from the point distributions (third row: using a coarse grid with spacing *h* = 0.1, fourth row: using a finer grid *h* = 0.02). As one can see in figure 8, while the number of points to throw to approximate the continuum density does not play a major role for high-density clustered distributions, it becomes critical for approximating more homogeneous distributions (compare left and right columns). Moreover, high-density clusters require the use of a fine enough grid to correctly recover the initial distribution (compare third and fourth rows in the left column), while smoother distributions are better approximated using a large number of agents and coarse grids (third row of the left columns). These first results highlight the necessity for adapting the numerical grid used to compute the density of agents from the discrete model if one hopes to have a consistent quantifier to compare with the continuum model.

A natural choice for comparing point clouds and continuum densities is to choose a Cartesian grid for both models, and compute the density of the individual agents using for instance a PIC method [37]. However, the choice of grid points spacing is critical, as it depends on the profile of the distribution as well as on the number of particles present in the computational domain: highly clustered agent distributions require fine meshes to enable the characteristics of the small and concentrated agent clusters to be captured, while more homogeneous agent distributions require coarser grids to allow the capture of larger patterns (figure 8). To be efficient, the grid spacing must, therefore, account for the characteristic size of the continuum structures that can be captured with a finite number of individual points. As we want to compare a continuum model with a discrete one, we will use the continuum simulations as a reference. Our goal here is to find the optimal Cartesian mesh on which a continuum density *ρ*^Δ*x*^(*x*, *t*) would be best represented by a cloud of *N* points, for *N* given. Note that the continuum density *ρ*^Δ*x*^(*x*, *t*) is itself already discretized on a Cartesian mesh with spacing Δ*x*, because it corresponds to a solution of the discretized continuum model.

Given a continuum density profile *ρ*^Δ*x*^(*x*, *t*)—discretized on a Cartesian mesh $\Omega_{\Delta x}\subset \Omega$ with grid spacing Δ*x* = 1/*N*_*x*_ in each direction—we first throw *N* individual points $(y_{1},\ldots ,y_{N})\in \Omega$ according to the distribution *ρ*^Δ*x*^(*x*, *t*). We now denote by $\rho_{\mathrm{PIC}}^{h}(y_{1},\ldots ,y_{N})$ the density of the individual points $\left(y_{1},\ldots ,y_{N}\right)$ computed on a Cartesian mesh of spacing *h* > 0 using a PIC method, and $\Pi^{\Delta x}(\rho_{\mathrm{PIC}}^{h})$ its linear interpolation on the initial mesh $\Omega_{\Delta x}$. We aim at finding the optimal grid spacing *h* minimizing the *L*^2^ distance between the initial continuum density *ρ*^Δ*x*^(*x*, *t*) and its approximation by *N* individual points

$$\tilde{h}=\underset{h}{\text{argmin}}\;\|\rho^{\Delta x}-\Pi^{\Delta x}(\rho_{\mathrm{PIC}}^{h}(y_{1},\ldots ,y_{N})){\|}_{\ell^{2}(\Omega_{\Delta x})},$$

where $\|.{\|}_{\ell^{2}(\Omega_{h})}$ denotes the discrete *l*^2^ norm on a Cartesian mesh $\Omega_{h}$

$$\|\rho^{h}{\|}_{\ell^{2}(\Omega_{h})}=h^{2}\sum_{i=1}^{N_{x}}\sum_{\;j=1}^{N_{x}}|\rho^{h}(x_{i},y_{j}){|}^{2}.$$

The optimal $\tilde{h}$ is computed numerically. It therefore corresponds to the best grid spacing one can hope for approximating a density *ρ*^Δ*x*^ with a set of *N* points. We therefore will use this quantity to compare a simulation of the continuum model with one of the discrete model performed with *N* agents. The discretized macroscopic density will be denoted by $\rho_{\mathrm{mac}}^{\tilde{h}}=\rho_{\mathrm{PIC}}^{\tilde{h}}(y_{1},\ldots ,y_{N})$, and the approximation from the discrete particle simulation will be denoted by $\rho_{\mathrm{mic}}^{\tilde{h}}$ (computed via the PIC method on a grid with spacing $\tilde{h}$). In the following section, we describe how to compare $\rho_{\mathrm{mac}}^{\tilde{h}}$ with $\rho_{\mathrm{mic}}^{\tilde{h}}$.

#### 5.1.2. Comparing discretized and discrete dynamics

**Figure 9.**
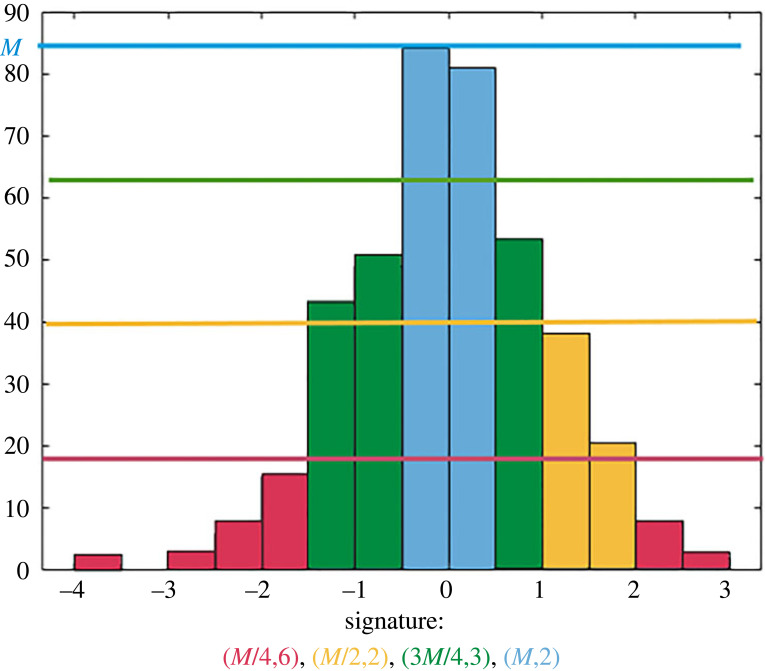
Example of the signature of a distribution with four bins, *M* = 85 and *N*_*x*_ = 13. The different colours represent the different compartments of the signature.

**Figure 10.**
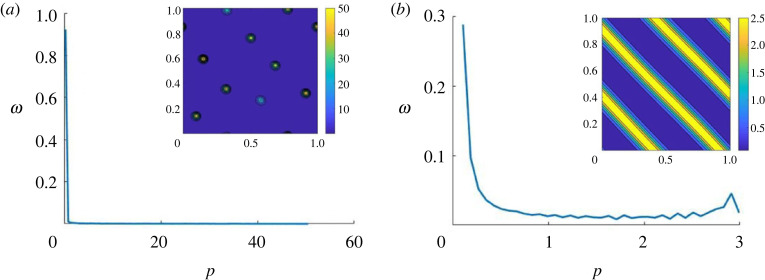
Histograms for simulations of figure 8 as defined in equation (5.1).

**Figure 11.**
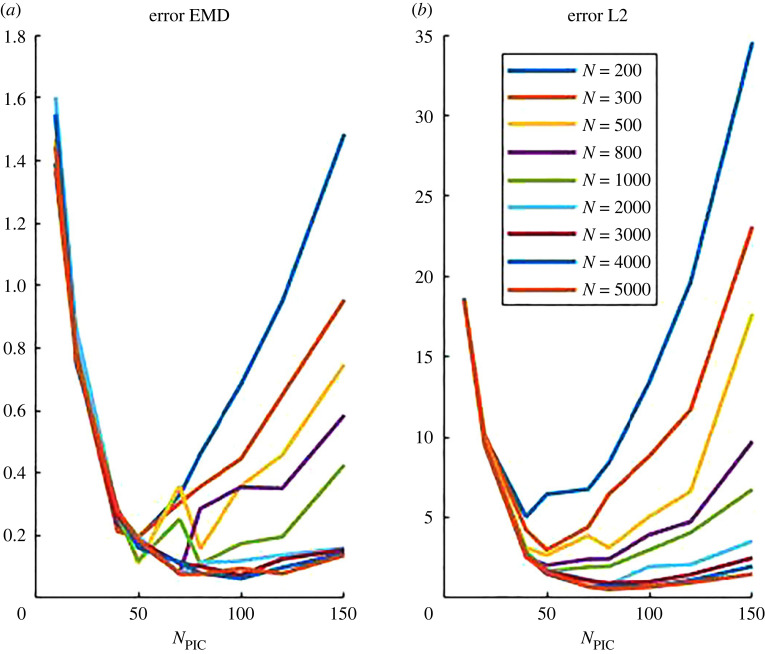
Error between the density profile of the continuum simulation (high-density clustered simulation of figure 8 left column) and its approximations using the procedure described in §5.1.1, as a function of the number of grid points for the PIC method *N*_PIC_ (horizontal axis) and different number *N* of discrete particles (see insert for correspondence between curve colour and *N*), using the EMD distance based on histograms (*a*) or the L2 distance based on point values (*b*).

The comparison between the discretized and the discrete dynamics will be done in several steps:

(Step 1) *Choosing the right distance to compare the micro- and macro-simulations:* we want to construct a quantifier enabling us to compute the distance between the two distributions $\rho_{\mathrm{mac}}^{\tilde{h}}$ and $\rho_{\mathrm{mic}}^{\tilde{h}}$ described in the previous section (solutions of the continuum and discrete models projected on a Cartesian mesh with spacing $\tilde{h}$). The first natural choice would be to use the discrete *L*^2^ norm as both quantities are defined on the same meshes. However, we need a quantifier independent of space translations, as there is no reason for the patterns of the discrete model to match exactly the locations of those of the continuum model at a given time. For example, if the discrete and continuum simulations produce band patterns with the same width and speed but not at the same positions, we still want to consider that the two solutions are very close to each other. Therefore, we propose here to use a Wasserstein-like distance.
  Inspired from [38], we choose to work with the earth movers distance (EMD). The EMD is based on the minimal cost that must be paid to transform one distribution into the other and relies on the solution to a transportation problem issued from linear optimization. As solving the transport problem in two dimensions is very costly, we ‘compress’/approximate the density distributions using their signatures (histograms).
(Step 2) *Construction of the signatures of the distributions:* given a density profile on a grid containing *N*_*h*_ = 1/*h* points in each direction (*ρ*_*ij*_), $i=1\ldots N_{h},j=1\ldots N_{h}$, the signature of *ρ*, $P[\rho ]=\{(p_{1},\omega_{1}),\ldots ,(p_{m},\omega_{m}))\}$ is defined as
  5.1 $$p_{k}=\frac{kM}{n_{b}}\;\mathrm{and}\;\omega_{k}=\sum_{i=1}^{N_{h}}\sum_{\;j=1}^{N_{h}}1_{\left[p_{k-1},p_{k}\right]}(\rho_{ij}),\;k=1\ldots n_{b},$$
  where *M* = ‖*ρ*‖_∞_ = max_*i*,*j*_ *ρ*_*ij*_ and the number of bins *n*_*b*_ has been chosen using the Freeman Diaconis rule, for which the bin width corresponds to 2 (IQR/*n*^3/2^), where IQR is the interquartile range of the data and *n* is the number of observations (in our case the number of grid points, *n* = 1/*h*^2^). We give in figure 9 a visual representation of computing the signature of a toy distribution with four bins and in figure 10 an example of the histograms of two simulations of the continuum model. Note that when computed on density distributions, the points *p*_*k*_ in each cluster correspond to local density values and the corresponding weights *ω*_*k*_ are the number of grid (spatial) points in which the density is comprised between the values *p*_*k*−1_ and *p*_*k*_.
(Step 3) *Definition of the EMD between two signatures:* following the lines of [38], we apply the following linear programming problem: Let $P=\{(p_{1},\omega_{1}),\ldots ,(p_{m},\omega_{m}))\}$ and $Q=\{(q_{1},v_{1}),\ldots ,(q_{n},v_{n}))\}$ be two signatures with *m* and *n* clusters represented by their representatives *p*_*k*_, *q*_ℓ_ and their respective weights *ω*_*k*_, *v*_ℓ_ for k=1…m,ℓ=1…n. We want to find a flow *F* = (*f*_*k*ℓ_) minimizing the overall cost
  $$W(P,Q,F)=\sum_{k=1}^{m}\sum_{\ell =1}^{n}d_{k\ell }f_{k\ell },$$
  where *d*_*k*ℓ_ is the ground distance matrix between clusters *p*_*k*_ and *q*_ℓ_
  $$d_{k\ell }=|p_{k}-q_{\ell }|.$$
  The minimization is made under the following set of constraints:
  5.2 $$f_{ij}\geq 0,\;1\leq i\leq m,1\leq j\leq n,$$
  5.3 $$\sum_{\;j=1}^{n}f_{ij}\leq \omega_{\;pi},\;1\leq i\leq m$$
  5.4 $$\sum_{i=1}^{m}f_{ij}\leq \omega_{qj},\;1\leq j\leq n$$
  5.5 $$\mathrm{and}\;\;\sum_{i=1}^{m}\sum_{\;j=1}^{n}f_{ij}=\min\left(\sum_{i=1}^{m}\omega_{\;pi},\sum_{i=1}^{m}\omega_{qj}\right).$$
  If we look at the signatures *P* and *Q* as a set of goods at given locations (represented by *p* and *q*) each with a given amount (represented by the weights *ω* and *v*), the EMD can be seen as a transportation problem consisting of finding the least expensive flow of goods from the suppliers to the consumers, where the cost of transporting a single unit of goods is given. Then, constraint (5.2) expresses that ‘supplies’ can be transported from *P* to *Q* only, while constraints (5.3), (5.4) limit the amounts of supplies that can be given by *P* to *Q* and that can be received from *Q* to *P*, respectively. The final constraint (5.5) expresses the fact that the total amount of mass transported must be optimal. Once this transportation problem is solved, the EMD between signatures *P* and *Q*, EMD(*P*, *Q*) is then defined as:
  $$\mathrm{EMD}(P,Q)=\frac{\sum_{i=1}^{m}\sum_{\;j=1}^{n}d_{ij}f_{ij}}{\sum_{i=1}^{m}\sum_{\;j=1}^{n}f_{ij}}.$$
  Rubner *et al.* proved in [38] that when the ground distance is a metric and the total weights of the two signatures are equal, the EMD is a true metric. Therefore, by considering the Euclidean distance as ground distance we can use the EMD as a valid dissimilarity measure between signatures. However, as two different density distributions may have the same signature, the EMD with (5.1) as signatures is a pseudo-metric. However, as shown in figure 10, the histograms between band like patterns and clustered state are very different distributions, making this pseudo metric suitable for measuring the dissimilarity between pattern types. Moreover, we check carefully in the next paragraph the validity of the EMD when it can be compared to the classical *L*^2^ distance.
(Step 4) *Validation of the pseudometric EMD.* In order to check the validity of the pseudometric constructed in this section, we aim to compare the efficiency of the EMD in cases where it can be compared to the classical *L*^2^ distance. More specifically, we use it to measure the dissimilarity between the density profile of the continuum simulation (high-density clustered simulation of figure 8 left column) and its approximation by a cloud of *N* points reconstructed on a grid, using the procedure described in the previous section. We show these dissimilarity measures in figure 11, as a function of the number of grid points for the PIC method (*N*_PIC_, horizontal axis of figures 11) and different numbers *N* of discrete particle (different curves), using the EMD distance based on histograms (*a*) or the *L*^2^ distance based on point values (*b*). As one can observe in figure 11, the EMD and the *L*^2^ norm are in good accordance. As previously observed in §5.1.1, both metrics show that for each number of particles used to approximate the continuum density distribution, there exists an optimal number of grid points for the PIC method which minimizes the distance between the initial density and its approximation by particles. As expected, this optimal value increases as the number of particles increases, suggesting that using a larger number of agents allows the use of finer grids which enables us to better capture the fine structures of the continuum density distribution. Moreover, this figure shows that the Wasserstein distance based on the EMD between density signatures seems to be a valid tool to compare density distributions.

In the next section, we present the numerical comparison between the discrete and continuum models.

**Remark 5.1.**

It is noteworthy that the patterns obtained are robust if we consider different realizations of the stochastic dynamics or slightly different initial conditions for the macroscopic dynamics (data not shown). In the following part of the paper, we will therefore compare the models based on only one realization of the microscopic dynamics.

### 5.2. Results

We aim to compare quantitatively the steady states of the discrete and continuum models in different regimes of the parameters, and study the influence of the number of agents for the discrete model *N* as well as the scaling parameter ϵ. We recall that the assumptions for the derivation of the continuum equations are given in §2.2.1. In particular, some of the parameters are scaled by a factor ε≪1 in the following way (denoting by a tilde the values used for discrete simulations):

5.6 $$\tilde{r_{R}}=\epsilon r_{R},\;\tilde{r_{A}}=\sqrt{\epsilon }r_{A},\;\tilde{d_{s}}=\frac{d_{s}}{\epsilon }\;\mathrm{and}\;\tilde{\nu }=\frac{\nu }{\epsilon }.$$

For all simulations, we consider the same number of agents and obstacles and set *M* = *N*, and we fix the values of $C_{\phi }=5$ (leading to *c*_0_ = 5.6) and *ζ* = 0.5. For each set of parameters, we use the method previously described in §5.1 to compare discrete and continuum simulations.

#### 5.2.1. Mild obstacle spring stiffness

**Figure 12.**
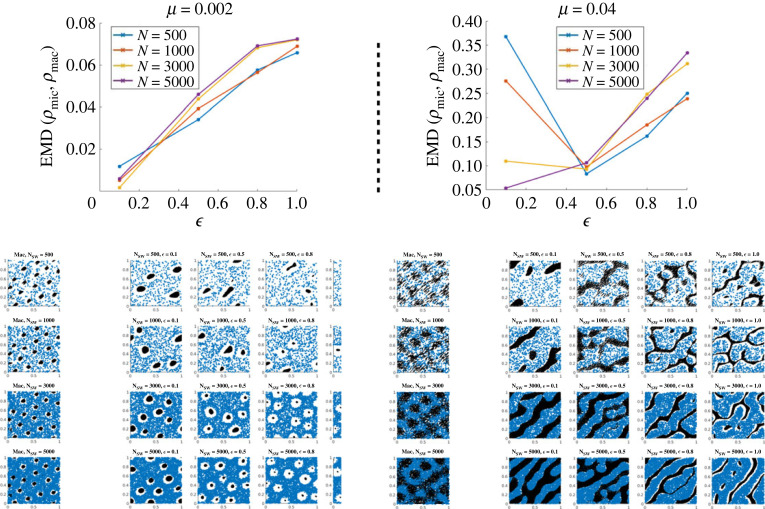
Comparison between the discrete and continuum simulations for mild obstacle spring stiffness *κ* = 100 and agent friction *ζ* = 0.5. Left figures: for weak agent-agent repulsion *μ* = 2 × 10^−3^, right figures, for *μ* = 4 × 10^−2^. Top figures: EMD between the approximated continuum density and the discrete one as a function of ϵ for different values of the number of agents *N*: *N* = 500 (blue curve), *N* = 1000 (red curve) and *N* = 3000 (yellow curve) and *N* = 5000 (purple curve). Bottom tables: simulations of the continuum model (left column), and of the discrete one for different values of ϵ: ϵ=0.1 (second column), ϵ=0.5 (third column), ϵ=0.8 (fourth column) ϵ=1 (last column). The different rows correspond to different number of agents for the discrete simulations as well as for the discretization of the continuum density (from top to bottom: *N* = 500, *N* = 1000, *N* = 3000, *N* = 5000).

In figure 12, we show the simulations obtained for mild obstacle spring stiffness *κ* = 100. The left panel is obtained for *μ* = 2 × 10^−3^ (corresponding to a bifurcation parameter *b*_*p*_ ≈ 0.036), and the right panel is for *μ* = 4.10^−2^ (corresponding to *b*_*p*_ ≈ 0.7, close to the stability threshold 1). Top figures show the EMD between the continuum and discrete solutions as a function of ϵ, for different number of agents used for the discrete simulations *N*: *N* = 500 (blue curve), *N* = 1000 (red curve), *N* = 3000 (yellow curve) and *N* = 5000 (purple curves). The corresponding simulations are shown below in tables: for each, the left column shows the simulations of the continuum model, and the next columns are simulations of the discrete model for different values of ϵ: ϵ=0.1 (second column), ϵ=0.5 (third column), ϵ=0.8 (fourth column), ϵ=1 (last column). The different rows of the tables correspond to different number of agents for the discrete simulations as well as for the discretization of the continuum density (from top to bottom: *N* = 500, *N* = 1000, *N* = 3000, *N* = 5000).

Figure 12 suggests that the discrete and continuum models are in quite good agreement in the case of week agent-agent repulsion (*b*_*p*_ ≪ 1, left panel), where both models are able to reproduce agent clusters, while their correspondence is more tenuous for stronger agent-agent repulsion (*b*_*p*_ close to the instability threshold, right panel), where the discrete dynamics seems to produce more trail-like patterns than the continuum model. For both regimes, however, we can observe a significant improvement of the discrete-continuum correspondence as ϵ decreases, suggesting that the continuum model becomes a good approximation of the discrete dynamics as ϵ goes to zero. Indeed, for weak agent-agent repulsion (left panel), we observe that decreasing ϵ is accompanied by an increase in the cluster sizes and a decrease of the distance between the boundary of the clusters and the obstacles, getting closer to the cluster types observed with the macroscopic dynamics. For stronger agent-agent repulsion (right panel), the clusters thicken as ϵ decreases and get closer to the continuum structures.

These observations are confirmed by the measurements of the EMD between the discrete and continuum agent distributions (top plots of figure 12). Indeed, one notes in the left panel that the distance between the two distributions decreases as the scaling parameter ϵ decreases, independently of the number of agents. Moreover, the top plot on the right panel shows that the discrete-continuum distance is larger for stronger agent-agent repulsion (*b*_*p*_ close to 1) compared to the case where *b*_*p*_ ≪ 1 (left panel). From the right figure, we also observe a strong dependency of the discrete-continuum distance as a function of the number of agents used in the discrete model. When the agent-agent repulsion is strong (or equivalently when *b*_*p*_ is close to 1), it becomes crucial to use a large number of individuals for the discrete simulations, while the number of agents does not seem to significantly impact the discrete-continuum agreement in regimes favouring the apparition of small and dense clusters (small agent-agent repulsion or equivalently small *b*_*p*_).

**Figure 13.**
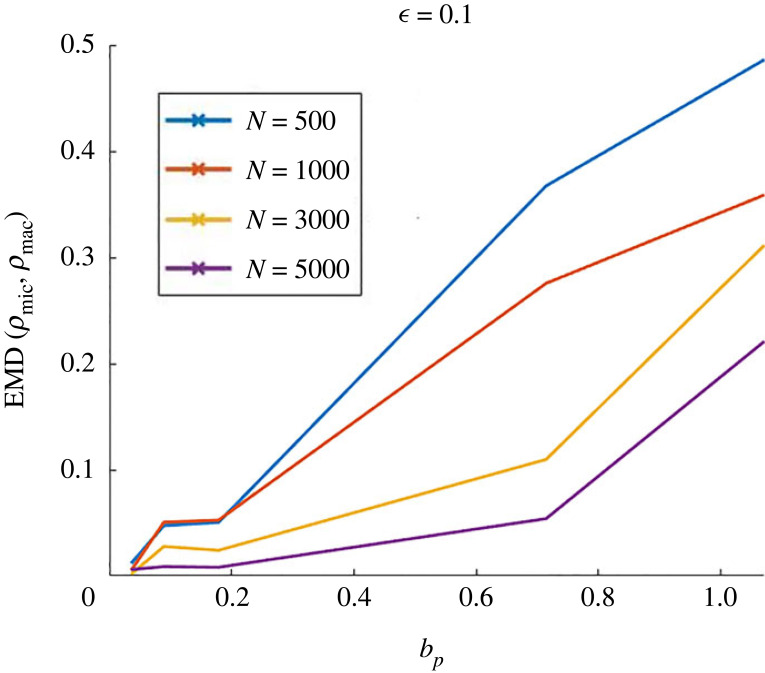
EMD between the approximated continuum density and the discrete one as a function of *b*_*p*_ for *κ* = 100, *ζ* = 0.5 and ϵ=0.1, and for different values of the number of agents *N*: *N* = 500 (blue curve), *N* = 1000 (red curve) and *N* = 3000 (yellow curve) and *N* = 5000 (purple curve).

These first observations tend to suggest that the choice of the number of agents in the discrete setting seems to depend both on the choice of ϵ and on the regime of parameters. In order to give more insights into the influence of *N* and *b*_*p*_ on the discrete-continuum match, we plot in figure 13 the EMD between the discrete and continuum models as a function of *b*_*p*_ (by changing the value of *μ* for fixed *κ* = 100), having fixed ϵ=0.1 and for different *N*:*N* = 500 (blue curve), *N* = 1000 (red curve) and *N* = 3000 (yellow curve) and *N* = 5000 (purple curve).

As one can see in figure 13, the discrete-continuum distance increases with *b*_*p*_ independently of the number of agents *N*, suggesting indeed that the discrete and continuum models are closer far from the instability threshold. As the agent-agent repulsion increases (increasing values of *b*_*p*_), the number of agents used in the discrete simulations has increasing influence on the match between the discrete and continuum simulations. These results suggest that large agent clusters with low density are better captured by a large number of agents.

#### 5.2.2. Strong obstacle spring stiffness

**Figure 14.**
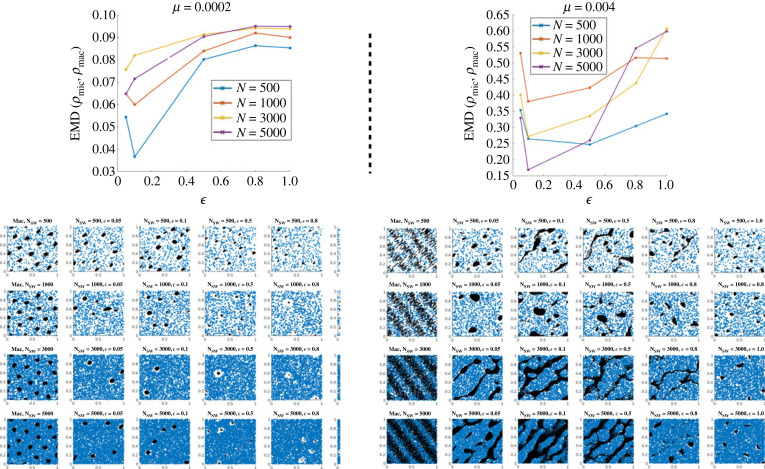
Comparison between the discrete and continuum simulations for strong obstacle spring stiffness *κ* = 1000 and agent friction *ζ* = 0.5. Left figures: for weak agent-agent repulsion *μ* = 2 × 10^−4^, right figures, for *μ* = 4 × 10^−3^. Top figures: EMD between the approximated continuum density and the discrete one as function of ϵ for different values of the number of agents *N*: *N* = 500 (blue curve), *N* = 1000 (red curve) and *N* = 3000 (yellow curve) and *N* = 5000 (purple curve). Bottom tables: simulations of the continuum model (left column), and of the discrete one for different values of ϵ: ϵ=0.05 (second column), ϵ=0.1 (third column), ϵ=0.5 (fourth column) ϵ=0.8 (fifth column) and ϵ=1 (last column). The different rows correspond to different number of agents for the discrete simulations as well as for the discretization of the continuum density (from top to bottom: *N* = 500, *N* = 1000, *N* = 3000, *N* = 5000).

Here, we aim to study the discrete-continuum agreement for strong obstacle spring stiffness *κ* = 1000. In figure 14, the left panel is obtained for *μ* = 2 × 10^−4^ (corresponding to a bifurcation parameter *b*_*p*_ ≈ 0.036), and the right panel is for *μ* = 4 × 10^−3^ (corresponding to *b*_*p*_ ≈ 0.7, close to the stability threshold 1). Top figures show the EMD between the continuum and discrete solutions as a function of ϵ, for different number of agents used for the discrete simulations *N*: *N* = 500 (blue curve), *N* = 1000 (red curve), *N* = 3000 (yellow curve) and *N* = 5000 (purple curves). As in the previous section, the corresponding simulations are shown below in tables: for each, the left column shows the simulations of the continuum model, and the next columns are simulations of the discrete model for different values of ϵ: ϵ=0.05 (second column), ϵ=0.1 (third column), ϵ=0.5 (fourth column) ϵ=0.8 (fifth column) and ϵ=1 (last column). As before, the different rows of the tables correspond to different number of agents for the discrete simulations as well as for the discretization of the continuum density (from top to bottom: *N* = 500, *N* = 1000, *N* = 3000, *N* = 5000).

For strong obstacle spring stiffness *κ* = 1000, we again observe that the discrete and continuous models are in good agreement far from the instability threshold (left panel), where both models reproduce clusters, while the agreement between the two models worsens for stronger agent-agent repulsion (right panel), where the discrete system fails to reproduce the travelling band patterns observed with the continuum model. Again, the discrete-continuum agreement improves as ϵ decreases: for low agent-agent repulsion the discrete pattern sizes converge to those of the continuum model as ϵ decreases (from right to left in the left panel), and for strong agent-agent repulsion (last rows of the right panel), decreasing ϵ induces a phase transition between clustered states and trail-like agent patterns, closer to the formation of bands.

It is noteworthy that for small agent-agent-repulsion (*b*_*p*_ ≪ 1, left figure), the agreement between the discrete and continuum dynamics seems to be better when using fewer agents in the discrete model, independently of the value of ϵ (compare purple and blue curves on the left panel), while close to the instability threshold (*b*_*p*_ close to 1, right figure) the choice of *N* seems to be related to ϵ: the discrete-continuum error decreases when using larger *N* for small ϵ, smaller *N* for larger ϵ.

#### 5.2.3. Summary of observations

We conclude that the continuum equations are a good approximation of the discrete dynamics in the limit of small rescaling parameter ε, as long as the agent-agent repulsion *μ* is small enough (i.e. in a parameter regime far from the instability threshold, *b*_*p*_ ≪ 1). On the contrary, the trend is less apparent when *μ* gets closer to the instability threshold μ* (corresponding to *b*_*p*_ = 1). In particular, when $\mu \approx \mu_{*}$, the rescaling factor ε can act as a phase transition parameter between different types of patterns (right panel, figure 14). This phase transition is due to the fact that the instability condition is given by (4.1). Indeed, the presence of *μ* in this formula hints at the fact that at the discrete level the agent-agent repulsion potential *ψ* plays a key role in determining the patterns that emerge. Therefore, it is no wonder that by rescaling the value of agent-agent repulsion radius ${\tilde{r}}_{R}=\varepsilon r_{R}$ (and therefore changing the value of *ψ*) the shape of the patterns also changes. However, the smaller the *μ* the less relevant the role of *ψ*, thus the predictions of the continuum simulations become more robust.

There is also another important factor to take into account: the continuum dynamics just gives averaged behaviour of the discrete dynamics. If there is a wide variability in the discrete dynamics, due to its intrinsic stochasticity, then the average behaviour will not be able to represent well particular realizations of the discrete dynamics. It seems that closer to the boundary of the instability region ($\mu \approx \mu_{*}$) this variability is larger.

## 6. Discussion

In this article, we have investigated a model for collective dynamics in an environment filled with obstacles that are tethered to a fixed point via a spring. The model was first introduced in [22]. In particular, the paper has presented the following novelties:

(i) phase diagram of the continuum equations in dimension 2;
(ii) a linear stability analysis of constant solutions;
(iii) method to discriminate between different types of patterns that has been used to compare quantitatively the relation between discrete and continuum simulations; and
(iv) a more extensive phase diagram of the discrete dynamics that has allowed us to identify two new types of patterns with respect to [22] (honey comb structures and pinned cluster states).

The continuum description captures well the behaviour of the system when it is comprised of a large number of agents and obstacles, and involves huge computational savings compared with the simulation of the discrete system. Comparing discrete and continuum simulations is in general not straightforward. We have proposed a method to compare the two types of solutions to investigate in which parameter regime they are in good correspondence. This parameter regime includes the assumptions made for the derivation of the continuum equations in §2.2.1: the spring stiffness must be large *κ* ≫ 1, the number of agents and obstacles must be large *N*, *M* ≫ 1, the scaling parameter ε≪1 (see (5.6) for the rescaled parameters) must be small. However, we require one more condition to have a good correspondence between discrete and continuum dynamics: the agent-agent repulsion intensity *μ* must be much smaller than the critical value $\mu_{*}$, which is at the threshold of the instability condition (4.1). For values closer to $\mu_{*}$, the intrinsic variability of the system is too large to be described just with the averaged behaviour that captures the continuum equations.

This work has also showcased the impact of the environment in pattern formation in collective dynamics. The phase diagrams of both discrete and continuum dynamics show that the feedback interactions between agents and obstacles give rise to a rich variety of patterns. In particular, we have observed that trails, travelling bands, moving clusters, uniform configurations and other in-between patterns emerge. The fact that agents can modify their environment by moving the obstacles is fundamental to this pattern emergence. This can be clearly seen in the linear stability analysis where the instability condition (4.1) depends crucially on the agent-obstacle repulsion force *ϕ* which is the only interaction force between agents and obstacles, and on the spring stiffness *κ* which indicates the degree of mobility of the obstacles around their tethered positions.

As a prospective work, we would like to use the models investigated here to study the impact of the environment in collective dynamics under a different setup. Following previous works on the Vicsek model [32], where the authors study the phase transitions for kinetic models describing self-propelled particles interacting through alignment, we could consider different initial conditions to study the impact of initialization on the types of patterns, and study exhaustively the phase transitions features (stability, convergence rate and hysteresis). Another interesting setup is collective motion in a complex fluid. To investigate this, the idea is to couple the current model with a fluid model. Then the environment in which collective motion takes place will be the combination of the fluid with the obstacles. The idea of representing a complex fluid in this manner is similar to other existing models in the literature, such as the Oldroyd-B model that describes the visco-elasticity of fluids filled with spring dumbbells [39]. The coupling of the current discrete model with a fluid model will require a new derivation of the continuum equations and a new linear stability analysis to understand how the presence of the fluid impacts the dynamics and pattern formation.

Another extension of this work will investigate the impact in collective dynamics of an environment filled with a different type of obstacle (i.e. obstacles of a different nature than the ones considered in this work). For example, one can consider solid obstacles that are movable but are not tethered or have a particular shape (like elongated fibres).

## Data Availability

The datasets supporting this article have been uploaded as part of the electronic supplementary material, appendices A and B for more details [40].
